# Diacylglycerol O-acyltransferase 2, a Novel Target of Flavivirus NS2B3 Protease, Promotes Zika Virus Replication by Regulating Lipid Droplet Formation

**DOI:** 10.34133/research.0511

**Published:** 2024-10-24

**Authors:** Xiaotong Luo, Yunxiang Yuan, Xiaocao Ma, Xin Luo, Jiannan Chen, Cancan Chen, Xiaoyi Yang, Jinna Yang, Xuanfeng Zhu, Meiyu Li, Yang Liu, Ping Zhang, Chao Liu

**Affiliations:** ^1^ Key Laboratory of Tropical Diseases Control (Sun Yat-sen University), Ministry of Education, Guangzhou, Guangdong 510080, China.; ^2^ Department of Immunology and Microbiology, Zhongshan School of Medicine, Sun Yat-sen University, Guangzhou, Guangdong 510080, China.; ^3^ Department of Pathology, The First Affiliated Hospital of Sun Yat-sen University, Guangzhou, Guangdong 510080, China.; ^4^ Experimental Teaching Center, Zhongshan School of Medicine, Sun Yat-sen University, Guangzhou, Guangdong 510080, China.; ^5^ School of Biomedical Sciences, Li Ka Shing Faculty of Medicine, The University of Hong Kong, Hong Kong SAR, China.

## Abstract

Various lipid metabolism-related factors are essential for Zika virus (ZIKV) replication. In this study, we revealed a crucial role of diacylglycerol O-acyltransferase 2 (DGAT2) in ZIKV replication using a short hairpin RNA-based gene knockdown technique. The replication of ZIKV was significantly inhibited by DGAT2 depletion in multiple cell lines and restored by trans-complementation with DGAT2. Mechanistically, DGAT2 is recruited in the viral replication complex by interacting with non-structural (NS) proteins. Among them, both human and murine DGAT2s can be cleaved by NS2B3 at the ^122^R-R-S^124^ site. Interestingly, the cleavage product of DGAT2 becomes more stable and is sufficient to promote the lipid droplet (LD) formation independent of its enzymatic activity. This work identifies DGAT2 as a novel target of the viral protease NS2B3 and elucidates that DGAT2 is recruited by viral proteins into the replication complex, thereby playing a proviral role by promoting LD formation, which advances our understanding of host–flavivirus interaction.

## Introduction

The Zika virus (ZIKV) is an emerging arthropod-borne virus belonging to the *Flaviviridae* family. ZIKV infection typically causes mild symptoms, but it has been linked to severe neurological disorders like Guillain–Barre syndrome and microcephaly in fetuses [1,2]. Consequently, ZIKV infection poses a significant threat to global public health [3]. Despite this, specific vaccines and drugs for ZIKV infection are currently unavailable.

Numerous studies have highlighted a crucial role of lipid metabolism in flavivirus infection. For instance, in Dengue virus (DENV) infection, fatty acid synthetase (FASN) is recruited to the replication site, increasing fatty acid synthesis and promoting virus replication by interacting with NS3 [4]. Additionally, inositol-requiring enzyme 1α (IRE1α) has been implicated in promoting ZIKV replication by affecting stearoyl-coenzyme A desaturase 1 (SCD1)-mediated lipid metabolism [5]. However, many other lipid metabolism-related host factors in ZIKV replication remain to be identified.

Given the close association between flavivirus replication and lipid metabolism [6–8], we aimed to explore the interplay between lipid metabolism-related host factors and ZIKV. We selected 12 crucial lipid metabolism-related factors and screened their effects on ZIKV infection through siRNA-based silencing. Among them, silencing of diacylglycerol O-acyltransferase 2 (DGAT2) significantly inhibited ZIKV replication. DGATs, encompassing DGAT1 and DGAT2, serve as the pivotal rate-limiting enzymes responsible for catalyzing the final step in triglyceride biosynthesis [9]. A topological study reveals that 4 transmembrane domains (TMDs) of DGAT1 are located near the N terminus and its C terminus oriented toward the endoplasmic reticulum (ER) lumen [10,11]. In contrast, DGAT2 features 2 TMDs near its N terminus, orienting both the N and C termini toward the cytosol. Especially, the C terminus of DGAT2 contains a membrane binding domain (MBD) and a lipid droplet (LD) targeting domain (LTD) [12–14].

DGAT2, highly expressed in lipogenic tissues such as adipose tissue, liver, and small intestine, plays a vital role in lipid metabolism, muscle energy metabolism, and fat absorption. Mice lacking DGAT2 are almost devoid of triglycerides and die during the perinatal period [15]. DGAT2, localized specifically on the ER and LDs, efficiently channels triglycerides from ER to LDs for LD expansion [12]. The studies on DGAT2 mainly focused on lipid metabolism-related diseases, and there are limited reports on its involvement in viral replication. To date, DGAT2 has only been reported to promote the replication of severe acute respiratory syndrome coronavirus 2 (SARS-CoV-2).

In this report, we investigated the roles of DGATs in ZIKV replication by generating DGAT1- or DGAT2-knockdown cells. We found that DGAT2, but not DGAT1, significantly promotes flavivirus replication. Moreover, we demonstrated that ZIKV utilizes the viral protease NS2B3 to enhance the stability of DGAT2 by cleaving it at the ^122^R-R-S^124^ site. This cleavage of DGAT2 is beneficial to viral replication. Our study illustrates the mechanism by which DGAT2, a novel cleavage target of the flavivirus NS2B3 protease, is hijacked by ZIKV to inhibit its degradation by the proteasome pathway, subsequently acting as a proviral host factor during viral replication.

## Results

### The replication of ZIKV is reduced in DGAT2-knockdown cells

**Fig. 1.**
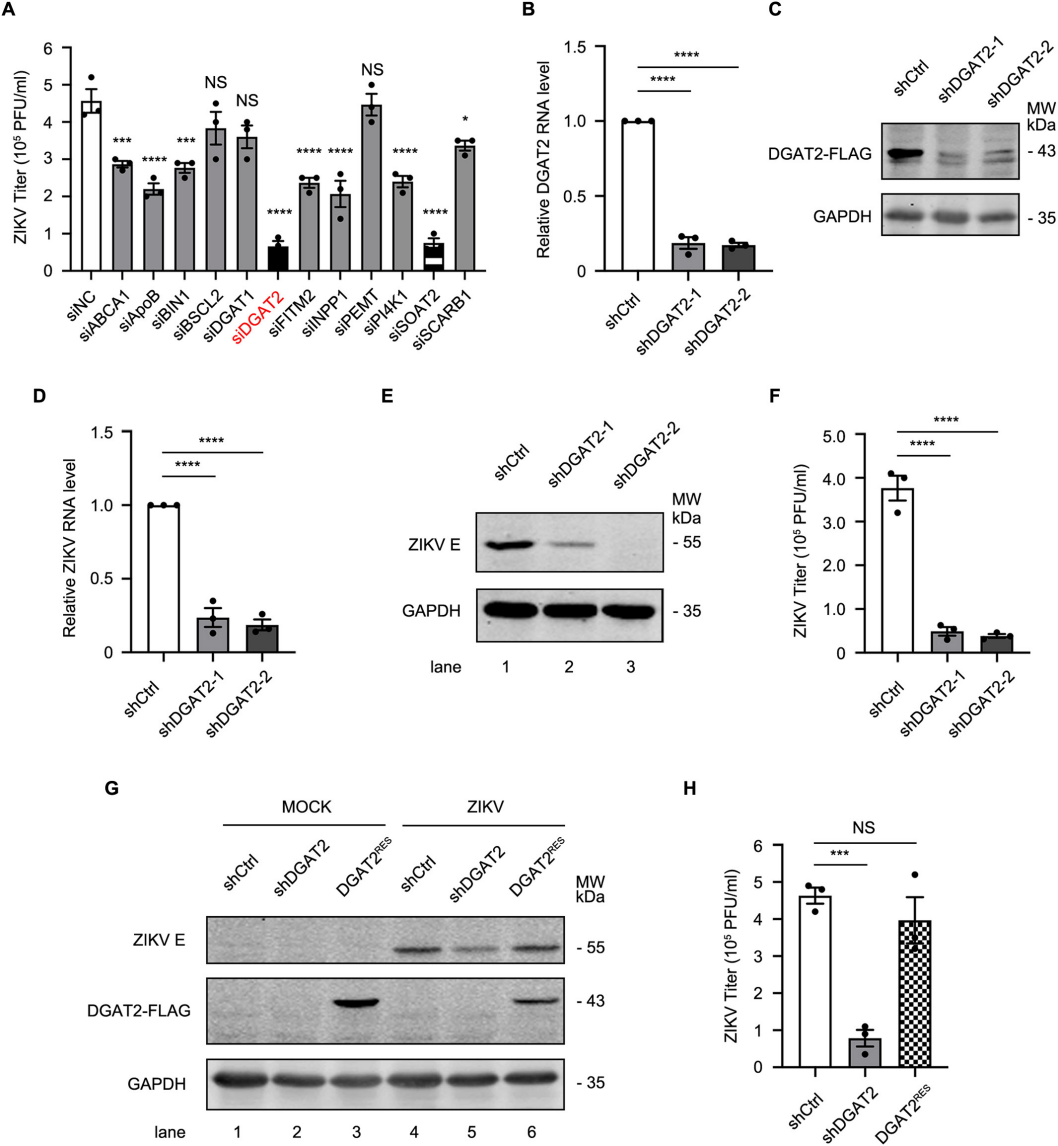
The replication of ZIKV is impaired in DGAT2-knockdown cells. (A) Screening of lipid metabolism-related genes associated with ZIKV replication. The Huh7 cells were infected with ZIKV at an MOI of 1 after 48 h of transfection with the control siRNAs or lipid metabolism-related gene-specific siRNAs and then harvested at 24 h after infection for plaque assay. (B and C) Confirmation of DGAT2-knockdown efficiency. The total RNAs of the control, DGAT2-knockdown-1, and DGAT2-knockdown-2 Huh7 cells were extracted for qRT-PCR assay (B). Due to the lack of useful commercial antibodies to detect the endogenous cellular DGAT2 levels, FLAG-tagged DGAT2-expressing plasmid was alternatively cotransfected with the DGAT2-shRNA-expressing plasmid. Knockdown efficacy of DGAT2-FLAG was determined by Western blotting using anti-FLAG antibody (C). (D to F) Effect of DGAT2 knockdown on ZIKV replication levels. The control and DGAT2-knockdown cells were infected with ZIKV at an MOI of 1 and harvested at 24 h after infection for qRT-PCR (D) or Western blotting (E). The E protein levels in Western blotting were normalized against GAPDH (E). The supernatants were collected for plaque assay (F). (G and H) Detection of viral replication levels. The control, DGAT2^KD^, and DGAT2^RES^ cells were infected with ZIKV (MOI = 1) and harvested at 24 h after infection for Western blotting (G). The supernatants were collected for plaque assay (H). Human *GAPDH* mRNA level was measured as an internal control for qRT-PCR. Data were shown as means ± SEM from at least 3 independent experiments. NS, no statistical significance; *P < 0.05; ***P < 0.001; ****P < 0.0001 (ANOVA with Dunnett’s multiple comparison test). GAPDH was probed as the loading control for Western blotting. Representative images of 3 independent experiments are shown.

Given the pivotal role of lipid metabolism in flavivirus infection, we conducted an siRNA screening of 12 lipid metabolism-related host factors associated with ZIKV replication (Table 1). Based on the siRNA data, we were particularly interested in DGAT2, as its silencing displayed the largest reduction of viral titer among the 12 genes (Fig. 1A). To further confirm the role of DGATs in ZIKV replication, DGAT2- or DGAT1-knockdown cells were generated. In the DGAT2-knockdown (DGAT2^KD^) cells, both DGAT2 mRNA and exogenous DGAT2-FLAG protein levels (the DGAT2 antibody did not work) were significantly decreased (Fig. 1B and C). Subsequently, ZIKV replication levels were analyzed. Compared with the control cells, the knockdown of DGAT2 led to a significant reduction in viral RNA levels (Fig. 1D). Similarly, the Western blotting result showed that the ZIKV E protein levels in DGAT2^KD^ cells were significantly lower than that in the control cells (Fig. 1E). As shown in Fig. 1F, the viral titers of DGAT2^KD^ cells were dramatically decreased by ~8-fold, suggesting that DGAT2 enhances the replication of ZIKV. In contrast, DGAT1 depletion did not affect the replication of ZIKV (Fig. S1).

**Table 1.** Twelve lipid metabolism-related genes mentioned in siRNA screening

Gene	Encoding protein	Function
*ABCA1*	Phospholipid-transporting ATPase	Translocates phospholipids from the cytoplasmic to the extracellular
*APOB*	Apolipoprotein B-100	Involves in lipid transport
*BSCL2*	Seipin	Plays a crucial role in the formation of LD
*BIN1*	Myc box-dependent interacting protein 1	Involves in the transport of lipids
*DGAT1*	Diacylglycerol O-acyltransferase 1	Synthesis of triglycerides
*DGAT2*	Diacylglycerol O-acyltransferase 2	Synthesis of triglycerides
*FITM2*	Acyl-coenzyme A diphosphatase	Required for maintaining ER structure and LD biogenesis
*INPP1*	Inositol polyphosphate 1-phosphatase	Participates in inositol phosphate metabolism
*PI4KA*	Phosphatidylinositol 4-kinase alpha	Acts on phosphatidylinositol in the production of the inositol-1,4,5, -trisphosphate
*PEMT*	Phosphatidylethanolamine *N*-methyltransferase	Catalyzes the steps of the methylation pathway for the biosynthesis of phosphatidylcholine
*SCARB1*	Scavenger receptor class B member 1	Mediates selective uptake of cholesteryl ether and HDL-dependent cholesterol efflux
*SOAT2*	Sterol O-acyltransferase 2	Catalyzes the formation of fatty acid cholesterol esters

To rule out the potential off-target effects of short hairpin RNAs (shRNAs) on virus infection, we generated DGAT2^RES^ cells by introducing human *DGAT2-FLAG* fusion gene with synonymous mutation in DGAT2-shRNA targeting sequence into DGAT2^KD^ cells and tested the changes in viral replication levels. The DGAT2 protein level was largely restored in DGAT2^RES^ cells (Fig. 1G), and ZIKV replication levels were successfully rescued (Fig. 1G and H). Furthermore, we examined the effect of DGAT2 on ZIKV replication in another 2 cell lines, namely, A549 and SNB19 cells. Similarly, a significant reduction of viral replication levels was observed in DGAT2^KD^ A549 and SNB19 cells (Fig. S2), indicating that the proviral effect of DGAT2 is not cell specific.

### DGAT2 promotes ZIKV replication at the viral RNA replication step

**Fig. 2.**
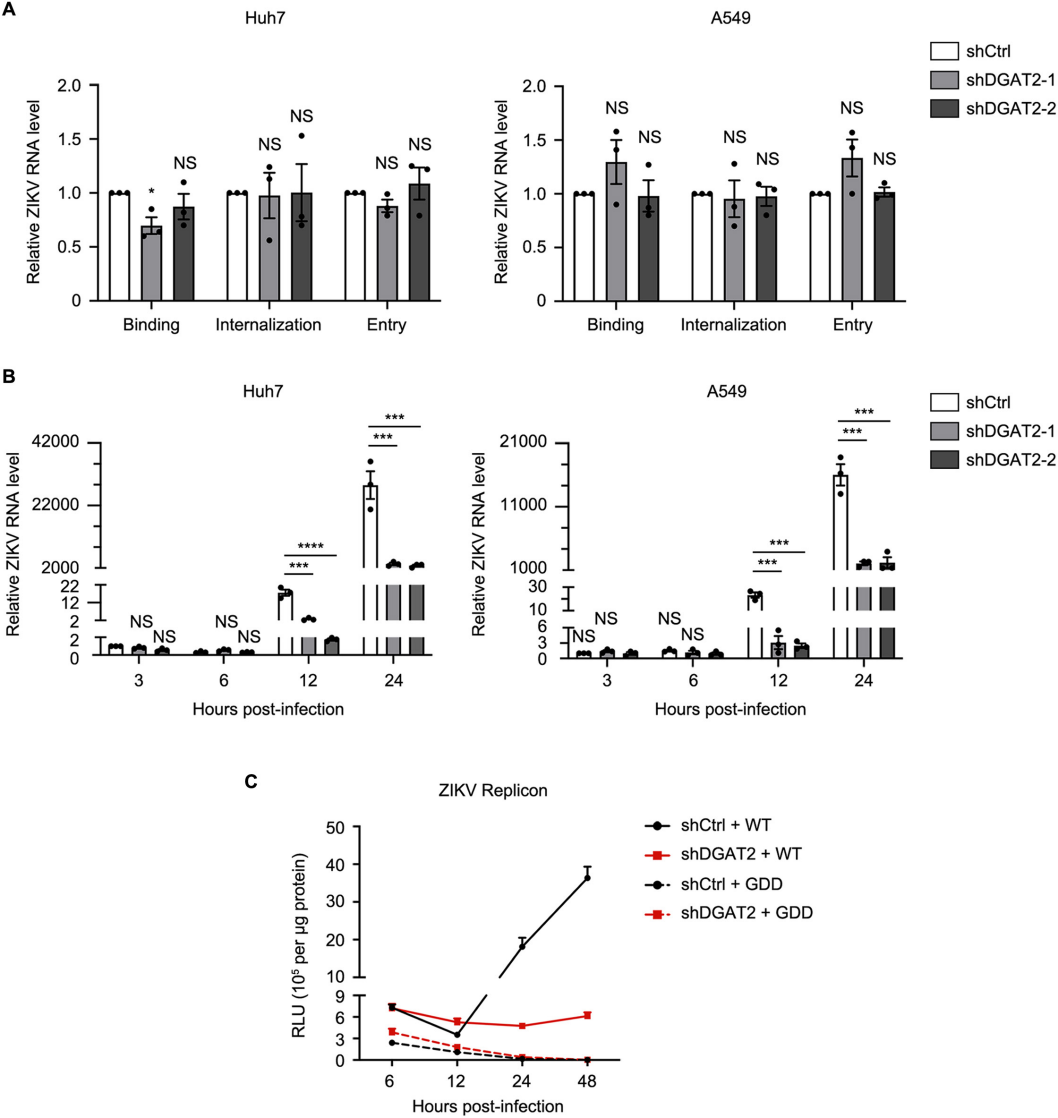
DGAT2 functions at ZIKV RNA replication step. (A) Effect of DGAT2 knockdown on viral entry. The control and DGAT2-knockdown cells were inoculated with ZIKV at an MOI of 1, followed by incubation on ice for 45 min (virion binding) or at 37 °C for 30 min (virion internalization) or at 37 °C for 60 min (virion entry). Cells were harvested for qRT-PCR. (B) Effect of DGAT2 knockdown on ZIKV RNA replication. The control and DGAT^KD^ cells were infected with ZIKV (MOI = 1) and collected at 3, 6, 12, and 24 h after infection. The viral RNA levels were measured by qRT-PCR. Human *GAPDH* mRNA level was measured as an internal control. (C) Replicon assay. The control and DGAT2^KD^ cells were transfected with ZIKV WT or GDD replicon RNAs, and harvested at indicated time points for luciferase assay. Data were shown as mean ± SEM of at least 3 independent experiments. *P < 0.05; ***P < 0.001; ****P < 0.0001 (ANOVA with Dunnett’s multiple comparison test).

Considering that both ZIKV RNA and protein levels were reduced by DGAT2 knockdown, we hypothesized that DGAT2 acts on the early step of ZIKV infection. First, the effect of DGAT2 on the viral entry step was tested. We inoculated the control, DGAT2^KD^, and DGAT2^RES^ cells with virus and then incubated the cells on ice for 45 min to detect virion binding, or at 37 °C for 30 min to analyze virion internalization or virion entry. As shown in Fig. 2A, the ZIKV RNA levels in the control, DGAT2^KD^, and DGAT2^RES^ cells were similar, indicating that DGAT2 is not involved in the viral entry process.

To detect whether DGAT2 affects the viral replication process, the ZIKV RNA levels from DGAT2^KD^ and control cells were compared at different time points. At 3 and 6 h after infection, ZIKV RNA levels were comparable in all groups; compared with the control group, ZIKV RNA levels in DGAT2^KD^ cells were decreased by ~7-fold at 12 h after infection, especially by ~9-fold at 24 h after infection (Fig. 2B), indicating that DGAT2 acts at the viral replication step.

To further distinguish which step, ZIKV protein translation or RNA synthesis, is affected by DGAT2, a viral replicon system was used. It is a subgenomic replicon that encodes 7 NS proteins and bears a *Renilla* luciferase reporter substituting the structural proteins [16]. The RNAs of wild-type (WT) ZIKV replicon and NS5 polymerase mutant replicon (GDD, which lacks RNA-dependent RNA polymerase activity) were in vitro transcribed and transfected into the control and DGAT2^KD^ cells. At 6 h after transfection, the luciferase values of WT and GDD replicon in the control and DGAT2^KD^ cells were similar, indicating that DGAT2 is not involved in the ZIKV protein translation. Significantly, the luciferase values of WT replicon in the DGAT2^KD^ cells were lower than those in the control cells at 24 h and 48 h after transfection (Fig. 2C), suggesting that DGAT2 regulates the ZIKV RNA replication step.

### DGAT2 is cleaved by flavivirus NS2B3 proteases

**Fig. 3.**
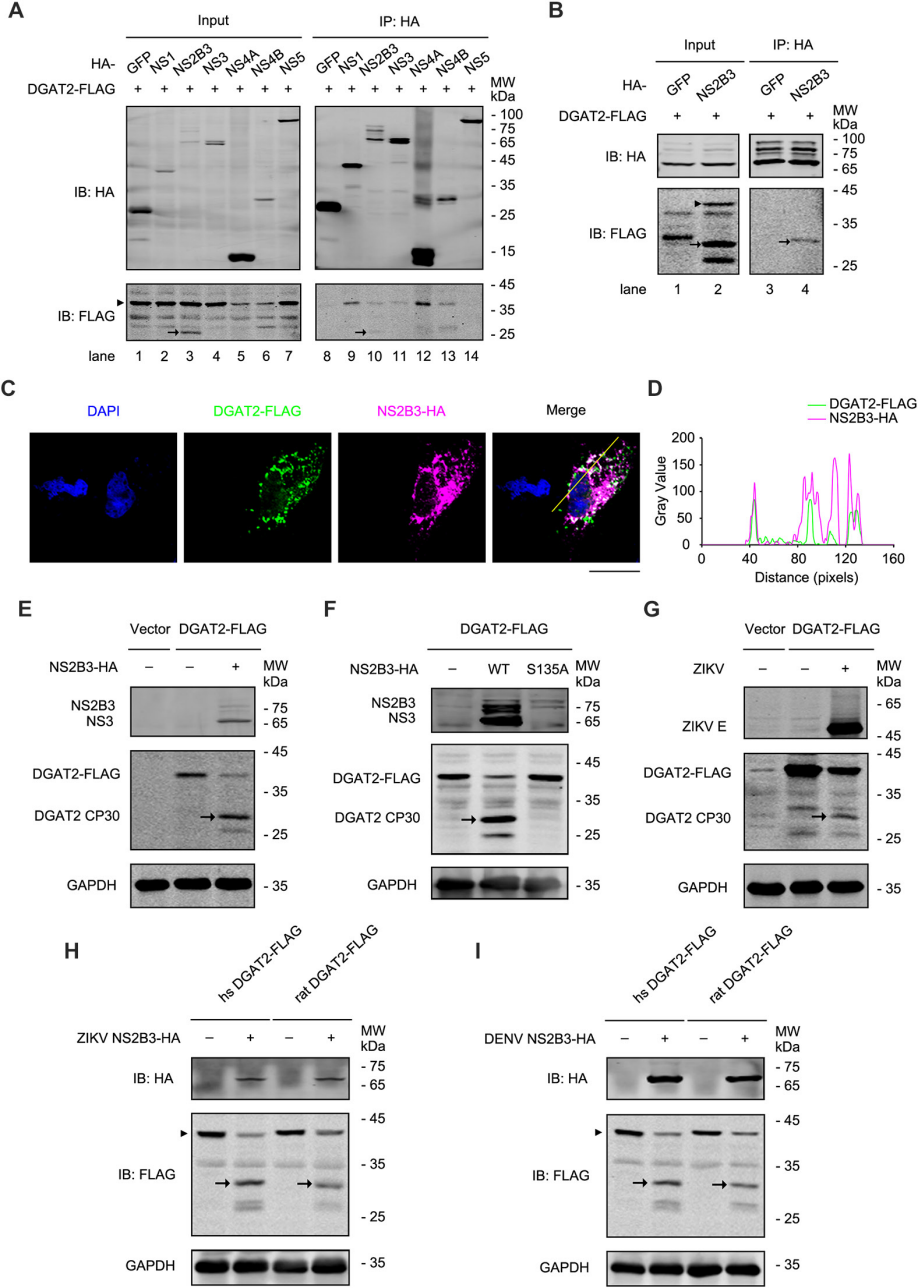
DGAT2 interacts with ZIKV NS proteins and is cleaved by protease NS2B3. (A) Co-IP assay of DGAT2 and ZIKV replication complex components in 293T cells. The 293T cells were cotransfected with plasmids expressing DGAT2-FLAG and ZIKV NS-HA proteins, and then whole-cell extracts were prepared for co-IP assay using anti-HA agarose beads at 24 h after transfection. Samples were detected by Western blotting using anti-FLAG and anti-HA antibodies. Green fluorescent protein (GFP)-HA was probed as the NC. IB, immunoblot. (B) Co-IP assay of DGAT2 and ZIKV NS2B3 in Huh7 cells. The Huh7 cells were cotransfected with plasmids expressing DGAT2-FLAG and ZIKV NS2B3-HA proteins. At 24 h after transfection, the whole-cell lysates were harvested and then prepared for co-IP assay using anti-FLAG antibody. GFP-FLAG was probed as the NC. (C) Subcellular localization of DGAT2 and ZIKV NS2B3. The Huh7 cells were cotransfected with plasmids expressing DGAT2-FLAG and NS2B3-HA. Anti-FLAG and anti-HA antibodies were used to indicate the subcellular localization of DGAT2 (green) and NS2B3 (magenta). Nuclei were stained with DAPI (blue). Scale bar, 100 μm. (D) Colocalization analysis using ImageJ software. (E and F) Immunoblot of Huh7 cells coexpressing DGAT2 and NS2B3 protein. Plasmids expressing FLAG-tagged human DGAT2 and HA-tagged ZIKV NS2B3 (E) or S135A mutant (F) were cotransfected in Huh7 cells for 24 h and then detected by Western blotting analysis. (G) Immunoblot of Huh7 cells expressing DGAT2-FLAG protein in the context of ZIKV infection. The Huh7 cells were transfected with the plasmid expressing DGAT2-FLAG for 24 h and then infected with ZIKV (MOI = 1). Cells were collected for Western blotting at 24 h after infection using anti-FLAG and ZIKV E protein antibodies. (E to G) DGAT2 CP30, DGAT2 cleavage product of ~30 kDa. (H and I) Immunoblot of Huh7 cells cotransfected with flavivirus NS2B3 and human/rat DGAT2-expressing plasmids. The plasmid expressing HA-tagged ZIKV NS2B3 (H) or DENV NS2B3 (I) and FLAG-tagged human DGAT2 or rat DGAT2 were cotransfected in Huh7 cells for 24 h, and then the DGAT2-FLAG-expressing level was detected by Western blotting assay. The DGAT2 cleavage fragment was marked by arrowhead, and full-length DGAT2-FLAG band was identified by black triangles. Representative images of 3 independent experiments are shown.

As many host factors are recruited into ZIKV replication complex by non-structural (NS) proteins, we proposed that DGAT2 probably participates in the formation of replication complex by interacting with viral NS proteins [17,18]. Thus, we detected the interaction between DGAT2 and NS proteins (NS1, NS2B3, NS3, NS4A, NS4B, and NS5). The result showed that except NS5, other viral NS proteins interact with DGAT2 to varying degrees (Fig. 3A).

Interestingly, we observed a smaller molecular mass band (~30 kDa) that represents a DGAT2 cleavage form in 293T cells co-expressing DGAT2 and ZIKV NS2B3 protease (Fig. 3A), implying that DGAT2 might be cleaved by NS2B3. Consistently, similar result was observed in NS2B3-DGAT2 coexpressing Huh7 cells (Fig. 3B). Moreover, NS2B3-HA (hemagglutinin) (magenta) and DGAT2-FLAG (green) were substantially colocalized in the cytoplasm (Fig. 3C and D). These data demonstrated that DGAT2 not only interacts with NS2B3 but also may be cleaved by ZIKV NS2B3.

To confirm that DGAT2 is a target of ZIKV NS2B3, we coexpressed DGAT2-FLAG and ZIKV NS2B3-HA in Huh7 cells (Fig. 3E). Consistent with the above results, an extra DGAT2 cleavage product of ~30 kDa (DGAT2 CP30) was detected. Next, the DGAT2-FLAG-expressing plasmid was cotransfected with the plasmid expressing WT NS2B3 or NS2B3-S135A mutant (lacking protease activity) [19]. As shown in Fig. 3F, the level of full-length DGAT2 was reduced and DGAT2 CP30 was only detected in the cells expressing WT NS2B3 but not NS2B3-S135A. Importantly, DGAT2 was also cleaved in the context of ZIKV infection (Fig. 3G). Therefore, these data suggested that ZIKV NS2B3 protease can cleave DGAT2.

Considering that human and murine DGAT2s are highly conserved (Fig. S3), we tested whether the rat DGAT2 is also sensitive to NS2B3. Expectedly, in NS2B3-expressing cells, the expression level of full-length rat DGAT2 was dramatically decreased, and a ~30 kDa cleaved product was detected (Fig. 3H). Furthermore, the cleavage effect of DENV NS2B3 on DGAT2 was also detected. Similarly, both human and rat DGAT2s can be cleaved effectively by DENV NS2B3 (Fig. 3I), indicating that DGAT2 is the target of flavivirus proteases.

### The ^122^R-R-S^124^ motif of DGAT2 is the cleavage site of NS2B3

**Fig. 4.**
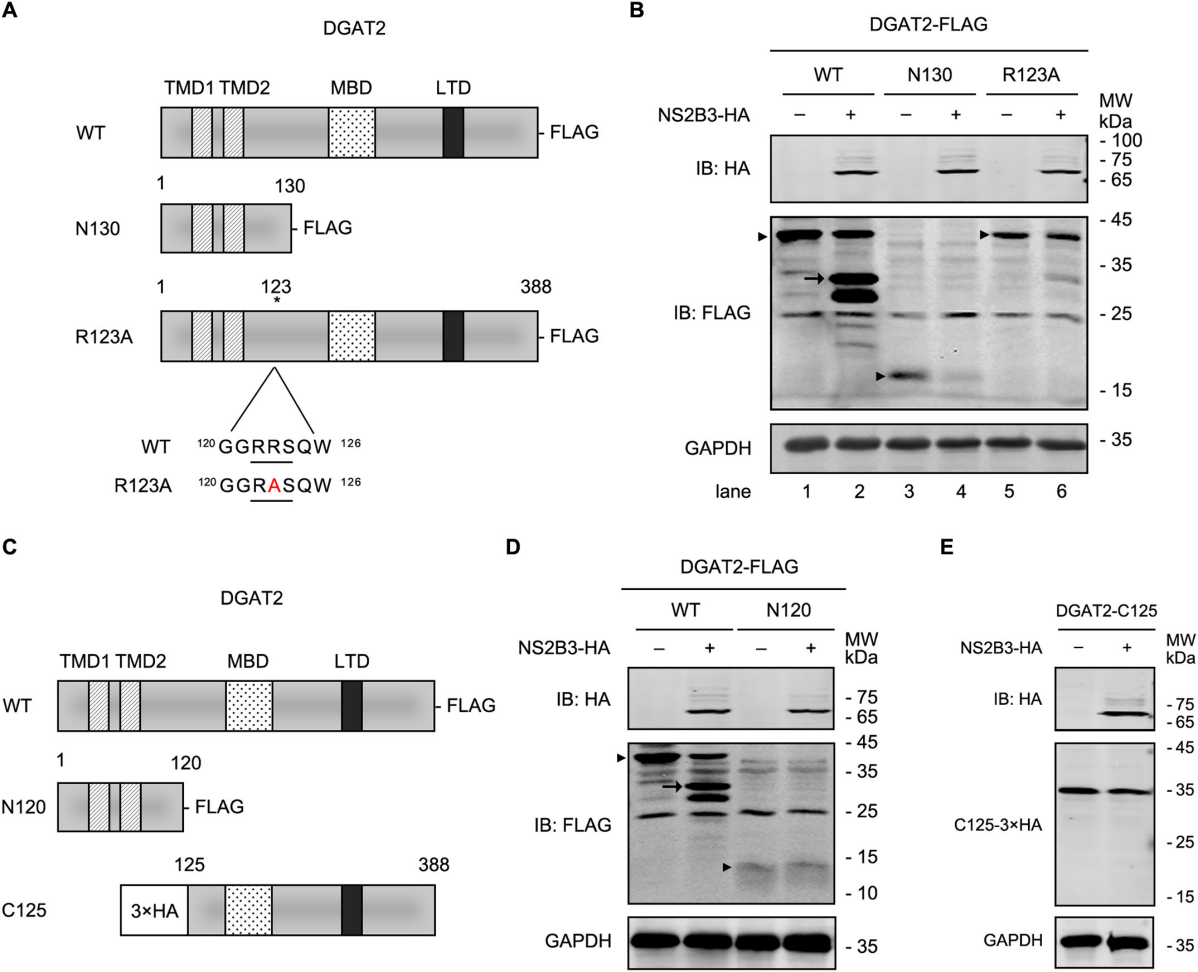
The ^122^R-R-S^124^ motif of DGAT2 is the cleavage site of ZIKV protease NS2B3. (A) Schematic diagram of WT and mutant DGAT2 constructs. DGAT2-N130 contains the first 130 aa of the N-terminal sequence. The 123rd arginine site was mutated into alanine in DGAT2-R123A, and the location of the mutated site was depicted by asterisk. TMD, transmembrane domain; MBD, membrane binding domain; LTD, LD targeting domain. (B) Immunoblotof Huh7 cells coexpressing ZIKV NS2B3 with DGAT2 mutants. The plasmid expressing ZIKV NS2B3-HA and DGAT2-WT-FLAG/N130-FLAG/R123A-FLAG was cotransfected in Huh7 cells for 24 h and then detected by Western blotting analysis. (C) Schematic diagram of full-length and truncated DGAT2 constructs. DGAT2-N120 contains the N-terminal 120 aa of DGAT2. 3×HA-DGAT2-C125 contains 125 to 388 aa of DGAT2fused with 3×HA at its N-terminus. (D and E) Immunoblottingof Huh7 cells cotransfected with plasmids encoding ZIKV NS2B3 and DGAT2 truncates. The plasmids expressing ZIKV NS2B3-HA and DGAT2-WT-FLAG/N120-FLAG (D) or 3×HA-DGAT2-C125 (E) were cotransfected in Huh7 cells for 24 h and then detected by Western blotting analysis. GAPDH was probed as an internal control for Western blotting. The DGAT2 cleavage fragment was marked by arrowhead, and FLAG-expressing bands were identified by black triangles. Representative images of 3 independent experiments are shown.

The general formula of ZIKV protease NS2B3 cleavage site is R/K/Q-R-↓-G/S [20,21]. Analysis of the DGAT2 sequence reveals 2 putative protease cleavage sites: the ^24^Q-R-S^26^ site and the ^122^R-R-S^124^ site. Based on the molecular weight of DGAT2 CP30, we predicted that the ^122^R-R-S^124^ motif in DGAT2 could be the most potential cleaved site of NS2B3. Thus, we generated 2 plasmids expressing DGAT2-N130-FLAG truncated protein that retains the N-terminal 130 amino acids (aa), and DGAT2-R123A-FLAG with predicted cleavage site mutated (Fig. 4A). Coexpression of NS2B3 decreased the level of DGAT2-N130 protein, suggesting that protease cleavage occurs within the C-terminal part of DGAT2-N130 (Fig. 4B). In addition, R123A mutation of the putative recognition site almost abolished the viral protease-dependent cleavage of DGAT2, supporting ^122^R-R-S^124^ as the main target site of ZIKV NS2B3 in DGAT2.

To determine whether another putative cleavage motif, ^24^Q-R-S^26^, is targeted by NS2B3, we constructed the plasmid expressing DGAT2 truncated protein (DGAT2-N120-FLAG) that retains the N-terminal 120 aa (Fig. 4C). NS2B3 expression did not lead to the reduction of DGAT2-N120 protein (Fig. 4D), indicating that the ^24^Q-R-S^26^ site is not the cleavage target of NS2B3. To be noted, a smaller band (~25 kDa) of DGAT2 was observed in the NS2B3-DGAT2 coexpressing cells, suggesting that other cleavage sites or start codon might be present downstream of ^122^R-R-S^124^ motif. First, we constructed an N-terminal 3×HA-tagged DGAT2 truncated protein (3×HA-DGAT2-C125) that retains 125 to 388 aa of DGAT2. As shown in Fig. 4E, the N-terminal HA tag of DGAT2-C125 was not cleaved by NS2B3, illustrating that no other cleavage sites were present downstream of ^122^R-R-S^124^. Then, we examined the DGAT2 protein sequence and found a methionine at site 166 aa downstream of the cleavage site R123, which might serve as another starting aa of this ~25 kDa band. Taken together, these results indicated that ZIKV NS2B3 cleaves DGAT2 at the ^122^R-R-S^124^ site.

### The 121-250 aa fragment of DGAT2 is essential for the DGAT2–NS2B3 interaction

**Fig. 5.**
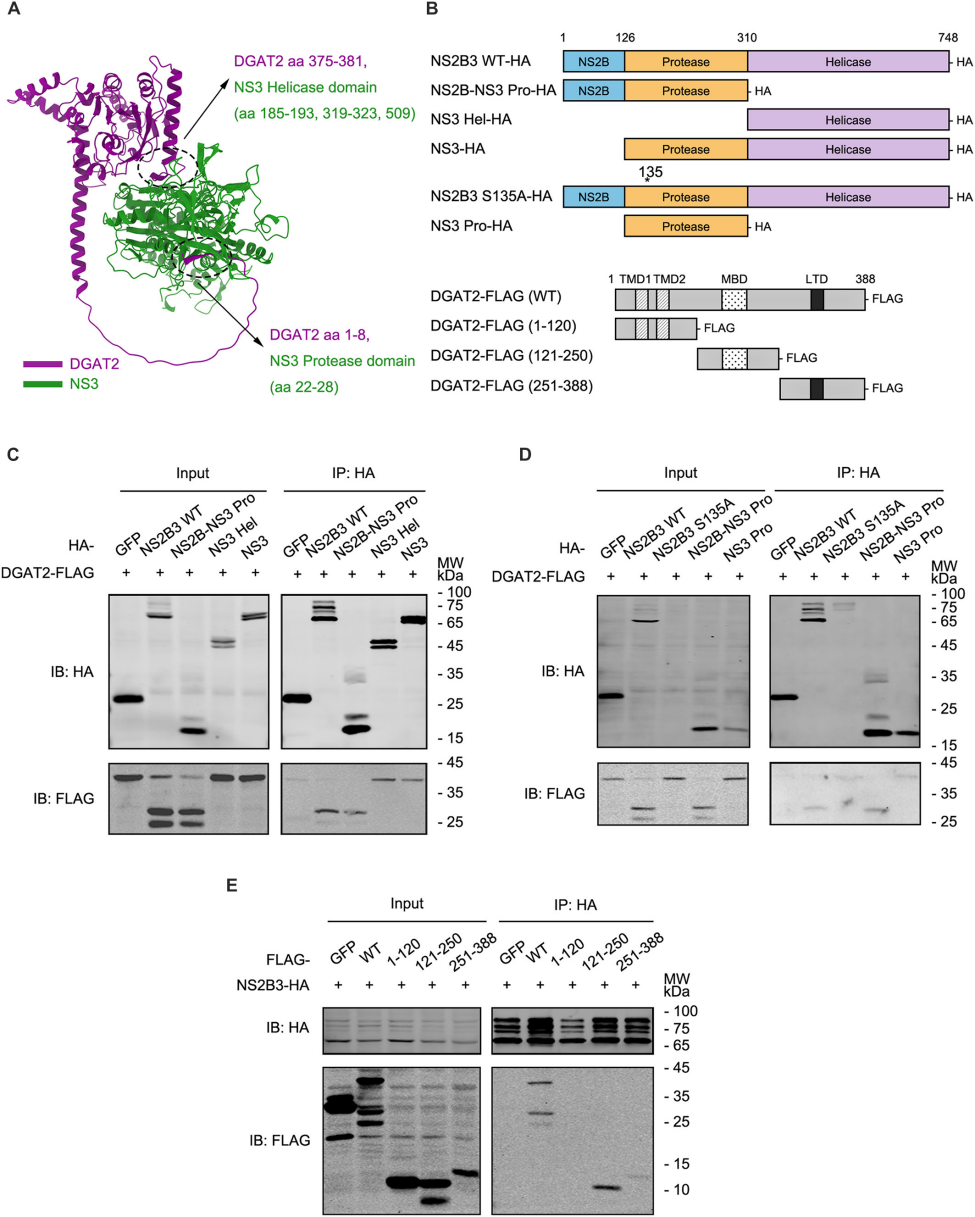
The 121-250 aa fragment of DGAT2 is essential for DGAT2 to interact with NS2B3. (A) Structure prediction analysis of human DGAT2 and NS3. The structure prediction was analyzed by AlphaFold 3 software, and the putative interaction sites in the structure were denoted. (B) Schematic illustration of constructs expressing truncated NS2B3 and DGAT2. (C and D) Co-IP assay to map the DGAT2-interacting domain of NS2B3. The Huh7 cells were cotransfected with plasmids expressing DGAT2-FLAG and different truncated proteins of NS2B3-HA. At 24 h after transfection, whole-cell lysates were harvested and then prepared for co-IP assay using anti-HA agarose beads. The protein levels were measured by immunoblot with anti-HA and anti-FLAG antibodies. (E) Co-IP assay to map the NS2B3-interacting domain of DGAT2. The Huh7 cells were cotransfected with plasmids expressing NS2B3-HA and different truncated proteins of DGAT2-FLAG. Immunoprecipitation and immunoblot analysis were performed similarly as in (C) and (D). GFP-HA (C and D) and GFP-FLAG (E) were used as the NC. Representative images of 3 independent experiments are shown.

In order to explore the interaction model between NS2B3 and DGAT2, we first predicted the NS2B3-DGAT2 interaction by AlphaFold 3 and found that there are putative interactions between NS3 and DGAT2. As shown in Fig. 5A, multiple parts of NS3 (distributed in its protease and helicase domains) were predicted to bind to DGAT2. To map the essential domain(s) of NS2B3 responsible for the interaction with DGAT2, we established a series of NS2B3 deletion mutants (Fig. 5B) [22,23]. The co-IP results showed that NS2B-NS3 protease (NS2B-NS3 Pro) fragment, NS3 helicase (NS3 Hel) fragment, and NS3 interacted with DGAT2 (Fig. 5C). It is worth noting that due to the low expression of the NS2B plasmid, we could not rule out the influence of NS2B on the interaction of NS3 with DGAT2.

As NS2B-NS3 Pro fragment interacts with DGAT2, we further explored whether the NS2B3 protease activity is required for the NS2B3-DGAT2 interaction. NS2B3-S135A and NS3 protease (NS3 Pro) mutants lacking protease activity were generated, and the binding of NS2B3-DGAT2 could be detected in the NS2B3-S135A or NS3 protease-expressing cells (Fig. 5D). These data indicated that both NS3 protease and helicase domains are required for the NS2B3-DGAT2 interaction, while the NS2B3 protease activity is dispensable.

To further detect the essential fragments of DGAT2 responsible for interacting with NS2B3, we detected the interaction between 3 FLAG-tagged truncates of DGAT2 (1 to 120 aa contains TMD; 121 to 250 aa contains MBD; 251 to 388 aa contains LTD) and NS2B3. As shown in Fig. 5E, the 121-250 aa fragment of DGAT2 interacted with NS2B3, whereas the N terminus of DGAT2 did not bind to NS2B3, demonstrating that the DGAT2–NS2B3 interaction is mainly mediated by the 121-250 aa fragment of DGAT2.

### The cleavage of DGAT2 is beneficial to ZIKV replication

**Fig. 6.**
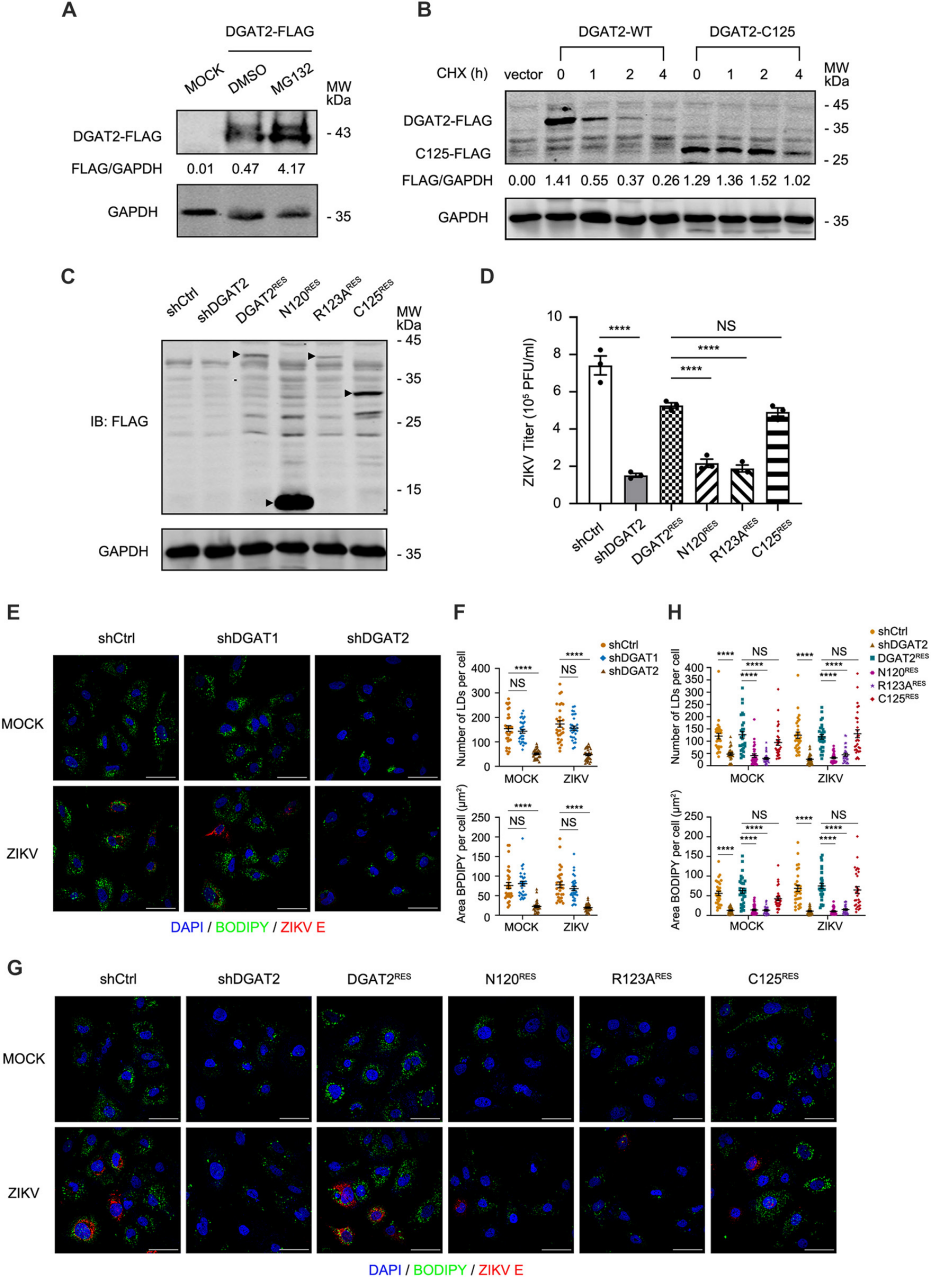
The cleavage product of DGAT2 (DGAT2-C125) is sufficient to promote ZIKV replication by regulating LD formation. (A) Inhibition of DGAT2 degradation by MG132. Huh7 cells expressing DGAT2-FLAG were treated with DMSO or MG132 for 10 h. The cell extracts were subjected to Western blotting analysis with anti-FLAG antibody. (B) Stability of DGAT2-C125 protein. The plasmid expressing DGAT2-FLAG or DGAT2-C125-FLAG was transfected into Huh7 cells for 24 h and then treated with the translation inhibitor CHX for the indicated times (0, 1, 2, and 4 h). The proteins were detected by Western blotting with anti-FLAG antibody. (C) Expression levels of DGAT2 truncated proteins. The Huh7 cells were transfected with the DGAT2-N120-FLAG-, DGAT2-R123A-FLAG-, or DGAT2-C125-FLAG-expressing plasmid. The cells were collected at 24 h after transfection and then detected by Western blotting with anti-FLAG antibody. The black triangles indicate the DGAT2 truncated protein bands. Representative images of 3 independent experiments are shown. GAPDH was analyzed as a loading control in (A) to (C). (D) Viral titers in DGAT2 truncated protein-expressing cells. The control, DGAT2^KD^, DGAT2^RES^, DGAT2-N120^RES^, DGAT2-R123A^RES^, and DGAT2-C125^RES^ Huh7 cells were infected with ZIKV (MOI = 1) and harvested at 24 h after infection for plaque assay. (E to H) LDs levels in cells. The control, DGAT1^KD^, DGAT2^KD^, DGAT2^RES^, DGAT2-N120^RES^, DGAT2-R123A^RES^, and DGAT2-C125^RES^ Huh7 cells were infected with ZIKV (MOI = 1) and then fixed at 24 h after infection (E and G). BODIPY staining was used to detect the formation of LDs (green). ZIKV E protein was stained by fluorescent antibody (red), and DAPI (blue) was used to indicate the nucleus (scale bars, 50 μm). The numbers and areas of LDs (at least 30 cells per sample) were analyzed by using ImageJ analysis software (F and H). Representative images of 3 independent experiments are shown. Data are shown as means ± SEM from at least 3 independent experiments. ****P < 0.0001 (ANOVA with Dunnett’s multiple comparison test).

Combining the findings that DGAT2 is rapidly degraded by the ubiquitin–proteasome pathway and its stability can be significantly enhanced after removal of its TMD domain [24,25] with our observations that the cleaved part of DGAT2 by NS2B3 contains its TMD domain, we hypothesized that the cleavage of DGAT2 might lead to an enhancement of its stability. As expected, the levels of DGAT2-FLAG protein were dramatically increased by MG132 treatment (Fig. 6A). Furthermore, full-length DGAT2 was rapidly degraded with a half-life of shorter than 1 h. In contrast, the degradation of DGAT2-C125 (DGAT2 cleavage protein, retains 125 to 388 aa) was much slower after treatment with the protein synthesis inhibitor cycloheximide (CHX) for 2 h (Fig. 6B), indicating that the NS2B3-mediated cleavage of DGAT2 enhances its stability.

Next, we further explored whether DGAT2-C125 confers a proviral activity. The plasmid expressing DGAT2-N120-FLAG, DGAT2-R123A-FLAG, or DGAT2-C125-FLAG was introduced into the DGAT2^KD^ cells. Compared with the control cells, ZIKV titers in DGAT2^RES^ and DGAT2-C125^RES^ cells were significantly rescued, but not DGAT2-N120^RES^ or DGAT2-R123A^RES^ cells (Fig. 6D). These results demonstrated that the cleavage product of DGAT2 is sufficient to support the viral replication.

### DGAT2 promotes ZIKV replication by regulating LD formation

As DGAT2 plays a role in the generation of LDs [14], which serve as an energy source for viral replication [26–28], we delved deeper into whether DGAT2 exerts its proviral function by influencing LD formation. The numbers and areas of LDs significantly decreased in DGAT2 ^KD^ cells, both with and without ZIKV infection, compared to control and DGAT1^KD^ cells (Fig. 6E and F), indicating that ZIKV-induced LD formation is hindered upon DGAT2 disruption. Considering that oleic acid (OA) is an important material of triglyceride (a component of LD) [5,29], we further examined the impact of LD production on ZIKV titers in DGAT2^KD^ cells with or without OA treatment. Consistent with the above result, the addition of OA dramatically reversed the decrease of LD accumulation and viral titers in the DGAT2^KD^ cells (Fig. S4). Moreover, the effects of DGAT2 truncated proteins on ZIKV-induced LD formation were assessed. Both the numbers and areas of LDs in the WT DGAT2^RES^ or DGAT2-C125^RES^ cells, but not DGAT2-N120^RES^ or DGAT2-R123A^RES^ cells, were dramatically higher than in the DGAT2^KD^ cells (Fig. 6G and H), suggesting that the C-terminal fragment of DGAT2 generated by NS2B3 cleavage is sufficient to promote LD formation. Taken together, these results indicated that DGAT2 promotes ZIKV replication by regulating LD formation.

Considering that the core contents of LDs are predominantly triglycerides, and DGAT2 is one of 2 key enzymes in triglyceride synthesis, we speculated that DGAT2 might affect ZIKV-induced LD formation by regulating intracellular triglyceride content. Thus, total triglyceride amounts in DGAT1^KD^, DGAT2^KD^, and DGAT1^KD^DGAT2^KD^ cells were measured. Individual knockdown of DGAT2 or DGAT1 in Huh7 cells did not affect total triglyceride amount, while double knockdown of DGAT1 and DGAT2 efficiently reduced the triglyceride amount (Fig. S5A), implying that the enzyme activities of DGAT2 or DGAT1 may compensate for each other. To further test whether the enzyme activity of DGAT2 is required for LD formation [30], we introduced DGAT2-C3A-FLAG (with 3 key enzyme active sites mutated: C87A/C96A/C312A) into DGAT2^KD^ cells (Fig. S5B) and detected changes in LDs upon ZIKV infection. The numbers and areas of LDs were comparable in control and DGAT2-C3A^RES^ cells, in both mock and ZIKV infected cells (Fig. S5C), indicating that the enzymatic activity of DGAT2 is not essential for the ZIKV-induced LD formation. Moreover, ZIKV titers in DGAT2-C3A^RES^ cells were not affected compared to those in control cells (Fig. S5D), suggesting that the pro-ZIKV function of DGAT2 is independent of its enzyme activity.

## Discussion

Host lipid metabolism has been linked to flavivirus replication and pathogenesis. Several lipid metabolism-related factors, such as SCD1 [5], SREBP2 [31], and AMPK [32,33], have been shown to participate in ZIKV replication. In this study, we identified DGAT2, a key rate-limiting enzyme catalyzing the final step of triglyceride biosynthesis, as a new ZIKV dependency factor.

First, our data showed that DGAT2, but not DGAT1, is a proviral factor for ZIKV replication. Interestingly, during the replication of hepatitis C virus (HCV) and rabies virus, DGAT1, rather than DGAT2, plays a proviral role [34,35]. These studies suggest that although DGAT1/DGAT2 plays an overlapping role in triglyceride biosynthesis, each of them has its unique functions. Structurally, DGAT1 and DGAT2 differ greatly as predicted by AlphaFold 3 (Fig. S1G and H). Particularly, DGAT2, but not DGAT1, can tether between ER and LD via its LTD, allowing the channeling of triglycerides from its synthesis site in ER to LD for the LD expansion [36]. To be pointed out, recent studies showed that DGAT1 inhibitor confers an anti-ZIKV activity in human primary placental cells [37], neuroblastoma cells, and mouse models [38]. However, they did not directly show the impact of DGAT1 on ZIKV infection. Considering that pharmacological inhibitors often possess broader off-target effects, our data utilizing gene knockdown technique are more specific, so we concluded that DGAT1 is dispensable in ZIKV replication.

Importantly, our study demonstrated that DGAT2 plays a critical role in viral RNA replication by interacting with viral NS proteins. Until now, several lipid metabolism-related factors have been demonstrated to interact with flavivirus NS proteins. FASN interacts with DENV NS3 through Rab18 to promote the synthesis of fatty acids [4,39]. Moreover, HCV NS5A could interact with DGAT1 [40], ARFRP1 [41], PLA1A [42], or PI4KIIIα [43], and then hijack them into the replication complex to promote viral replication. As all of these lipid metabolism-related factors are recruited in the replication complex to provide energy for viral replication and assembly, we propose that the interaction of DGAT2 with NS proteins might mediate the connection of the replication complex to LD, where viral replication and assembly take place.

Surprisingly, the association of DGAT2–NS2B3 leads to a cleavage of DGAT2, which dramatically enhances the stability of DGAT2. It is known that DGAT2 is unstable due to the ubiquitin–proteasome-mediated degradation, and becomes more stable upon removal of its N-terminal TMD domain. Our data also confirmed that compared to full-length DGAT2, the half-life of C-terminal part of DGAT2 is significantly prolonged by NS2B3 cleavage. Importantly, the C-terminal part of DGAT2 is sufficient to support the viral replication, and the DGAT2–NS2B3 interaction is independent of 1 to 120 aa of DGAT2, illustrating that the N-terminal part of DGAT2 is dispensable for ZIKV replication. So far, all reported cellular targets of flaviviral NS2B3, including STING [44,45], FAM134B [21], MFNs [46], and GSDMD [47,48], play the antiviral roles, and the NS2B3-mediated cleavage generally destroy their integrity and enable the virus to antagonize their functions. As far as we know, DGAT2 is the first proviral factor cleaved by NS2B3, and the cleavage results in the removal of its N-terminal part, which is not required for viral replication, therefore becoming more stable.

Furthermore, our work demonstrated that both human and rat DGAT2s can be cleaved by DENV/ZIKV NS2B3, indicating that DGAT2 may also be a proviral factor in murine. As human and murine DGAT2s show high homology with the same cleavage sites of NS2B3, it is not out of expectation that the murine DGAT2 can be cleaved by NS2B3. In contrast, the amino acids at the cleavage sites of STING and MFN-1 in human and mouse are different, so NS2B3 only cleaves human STING and MFN-1, but not mouse orthologous proteins. Collectively, the cleavage of host protein by the viral protease is highly dependent on the conservation of cleavage sites. Thus, in the process of species evolution, the mutation in the viral protease cleavage sites may enhance the resistance of species to viral infection.

Recently, the relationship between LDs and flaviviruses has been extensively studied. Flavivirus infection can significantly induce the formation of LDs, providing energy for viral replication and assembly [6,49]. In this study, we found that the ZIKV-induced LD formation in Huh7 cells was significantly inhibited by DGAT2 depletion. Intriguingly, knockdown of DGAT2 in Huh7.5 cells does not affect ZIKV titers (Fig. S6A to C), consistent with a previous work showing that knockdown of DGAT2/DGAT1 alone in Huh7.5 cells has no effect on LD formation [34]. As Huh7.5 cell line is a mutated derivative of Huh7 cells that confers thousands of mutations compared to Huh7 cells [50], it is not surprising to observe that the impact of DGAT2 depletion on the LD formation in Huh7 and Huh7.5 cells are distinct. These results further suggest that the proviral function of DGAT2 is closely related to LD formation.

The observations that knockdown of either DGAT1 or DGAT2 alone does not affect intracellular triglyceride amount, and the enzymatic activity of DGAT2 is not required for LD formation and ZIKV replication, suggest that the enzymatic activities of DGAT1 or DGAT2 could compensate for each other in terms of triglyceride synthesis. However, only DGAT2 can function as a tethering bridge between ER and LD to regulate the accumulation and expansion of LDs. Therefore, we propose that the proviral role of DGAT2 might be exerted through structural participation in the accumulation and expansion of LDs. Further evidence needs to be provided to elucidate the mechanism of LD accumulation and expansion affected by DGAT2 upon ZIKV infection. The changes in ZIKV-induced LD components, such as lipid types, molecular contents, location movement, and surface proteins, by LD isolation and purification should be evaluated in the future.

**Fig. 7.**
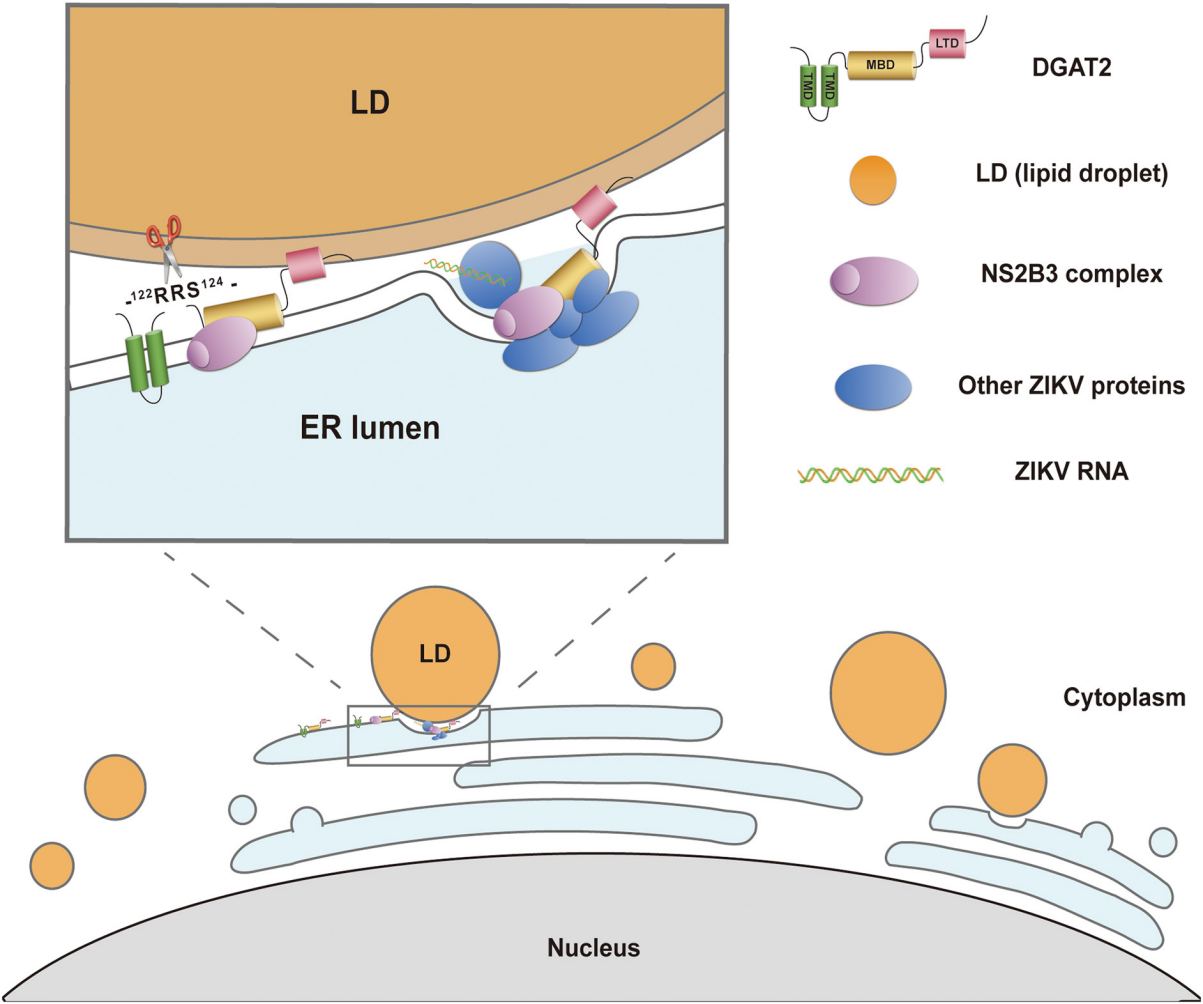
A proposed model to illustrate the mechanism of DGAT2 promoting ZIKV replication. In response to ZIKV infection, DGAT2 is cleaved by ZIKV protease NS2B3 and becomes more stable. Cleaved DGAT2 is subsequently recruited by NS proteins to the viral replication complex. DGAT2 may function as a tethering bridge between ER and LD, regulating LD accumulation to supply energy for ZIKV replication.

In summary, we unveiled a novel mechanism of the lipid metabolism-related factor DGAT2 promoting ZIKV replication (Fig. 7): Upon ZIKV infection, DGAT2 is cleaved by ZIKV protease NS2B3 to enhance its stability, providing more stable support for viral replication. Moreover, DGAT2 is hijacked to the viral replication site by NS proteins, where it functions as a tethering bridge between ER and LD, regulating LD accumulation to supply energy for viral replication. Further investigations on how DGAT2 regulates ZIKV-induced LD formation will be helpful for understanding the mechanisms of host–ZIKV interaction and also potentially provide a novel anti-ZIKV target for therapeutics.

## Materials and Methods

### Cell lines

Human lung carcinoma epithelial cells (A549), human hepatoma cells (Huh7 and Huh7.5), human glioblastoma cells (SNB19), human embryonic kidney cells (293T), and African green monkey kidney cells (Vero) were cultured in medium as described previously [5].

### Antibodies

Primary antibodies included anti-ZIKV envelope (E) (GTX-133314, GeneTex), anti-FLAG (PM020, MBL), anti-FLAG (20543-1-AP, Proteintech), anti-HA (M180-3, MBL), and anti-glyceraldehyde-3-phosphate dehydrogenase (GAPDH) (10494-1-AP, Proteintech). Secondary antibodies include IRDye 800 CW-conjugated anti-rabbit immunoglobulin G (IgG) (926-3221, LI-COR) and IRDye 680 CW-conjugated anti-mouse IgG (926-68020, LI-COR). Secondary antibodies used in immunofluorescence assay included goat anti-rabbit IgG antibody Cy3 conjugated (Sigma-Aldrich).

### Virus

The ZIKV H/PF/2013 strain (GenBank: KJ776791) was provided by Guangzhou Centers for Disease Control and propagated in Vero cells. When the cytopathic effect appeared, the supernatants were harvested and the cell debris was removed by centrifugation and filtration. Titers of virus stocks were determined and then stored at −80 °C.

### Virus infection

The cells were infected with ZIKV (MOI = 1). Both the supernatants and cells were collected at different time points for subsequent experiments.

### Virus titration

The supernatants of virus-infected cells were harvested for virus titration by standard plaque assay in Vero cells. In the plaque assay, serial 10-fold dilutions of each sample were prepared and 100 μl/well of the diluted virus was added into the 24-well plates. The cells were cultured at 37 °C for 1 h, and then the incubation medium was removed and cultured in the mixture of 2× Dulbecco’s modified Eagle’s medium (DMEM) (Invitrogen) and 2% methylcellulose (1:1) (Sigma). Visible plaques were counted at 4 to 5 d after infection. Cells were fixed with 10% formaldehyde for 1 h and then stained with 1% crystal violet.

### RNA interference

The sequences of 12 selected gene-specific siRNAs were designed and obtained by GUANGZHOU RIBOBIO. A control siRNA with scrambled sequence was used as a negative control (NC). Transfection was carried out with 40 nM siRNAs by using Lipofectamine 2000 reagent (Invitrogen) according to the manufacturer’s protocol. At 48 h after transfection, cells were harvested or used for further experiments.

### Quantitative RT-PCR

Total RNAs were extracted using TRIzol reagent (Invitrogen) according to the manufacturer’s instructions and then reverse transcribed using HI Script Q RT SuperMix (Vazyme). Quantitative reverse transcription polymerase chain reaction (qRT-PCR) was performed in a Bio-Rad CFX96 machine with SYBR Select Master Mix for CFX (Applied Biosystems), and the data were analyzed as described previously [51]. Primers were listed in Table 2.

**Table 2.** Sequences of primers used in qRT-PCR

Gene	Sequence (5′-3′)
*ZIKV NS1*	5F: GTCAGAGCAGCAAAGACAA
	3R: CAGCCTCCTTTCCCTTAACA
*DGAT1*	5F: CCTACCGCGATCTCTACTACTT
	3R: GGGTGAAGAACAGCATCTCAA
*DGAT2*	5F: GAATGGGAGTGGCAATGCTAT
	3R: CCTCGAAGATCACCTGCTTGT
*GAPDH*	5F: GCAAATTCCATGGCACCGT
	3R: TCGCCCCACTTGATTTTGG

### Western blotting

Cells were lysed in radioimmune precipitation assay lysis buffer as previously described [51]. Proteins were then separated on sodium dodecyl sulfate–polyacrylamide gel electrophoresis and transferred onto nitrocellulose membranes, followed by blocking in 0.1% phosphate-buffered saline–Tween 20 (PBST) buffer and incubating with primary antibody at 4 °C overnight. After incubation with secondary antibody, the Western blotting bands were visualized using an Odyssey IR imaging system (LI-COR).

### Plasmid construction and transfection

Fragments of WT DGAT2, DGAT2 rescued mutant, DGAT2-C3A-FLAG construct (the cysteine at positions 87, 96, and 312 of DGAT2 mutated into alanine), DGAT2-N130-FLAG, DGAT2-R123A-FLAG, DGAT2-N120-FLAG, DGAT2-C125-FLAG, 3×HA-tagged DGAT2-C125-FLAG, and several FLAG-tagged truncates of DGAT2 (1 to 120 aa, 121 to 250 aa, and 251 to 388 aa) were amplified with Huh7 cell Complementary DNAs (cDNAs) as template through PCR. Fragments of rat DGAT2-FLAG were amplified through PCR with the plasmid of pExpress-1-Dgat2 (rat) (P28822, obtained from MiaoLingBio, China) as template. The amplified fragments were purified and inserted into the Mlu I and Eco RI sites of pLVEF1a-IRES-Blast vector with FLAG fused with their C termini (N-terminal 3×HA-tagged in DGAT2-C125-FLAG). The cDNAs of Huh7 cell were used as template of the fragments of WT DGAT1 for PCR amplification. The amplified fragments were purified and inserted into the Eco RI and Hind III sites of pcDNA3.1 vector with FLAG fused with their C termini. ZIKV NS1-, NS2B3-, and NS3-expressing plasmids were amplified through PCR with viral cDNA as template. Several HA-tagged mutants of ZIKV NS2B3 (S135A, NS2B-NS3 Pro, NS3-Hel, and NS3-Pro) were amplified through PCR with the plasmid of ZIKV NS2B3 WT as template. Then, the PCR-amplified products were purified and inserted into pSG5 vector. ZIKV NS4A-, NS4B-, and NS5-expressing plasmids were obtained from G. Zhang’s team at Sun Yat-sen University. DENV NS2B3 was constructed as described previously [52]. The PCR primers used were listed in Table 3. All constructs were verified by DNA sequencing. Lipofectamine 2000 Reagent was used for cell transfection following the manufacturer’s instructions.

**Table 3.** Sequences of primers used in PCR amplification of gene fragments

Gene	Sequence (5′-3′)	
DGAT2-FLAG	5F: CGACGCGTATGAAGACCCTCATAGCCGCCTACTCC	
	3R: CGGAATTCTTACTTATCGTCGTCATCCTTGTAATCACCACCACCGTTCACCTCCAGGACCTC	
DGAT2-RES	5F: GGTGAAGACACACAACCTGCTAACGACGAGAAACTAC	
	3R: GGGGGTGGTATCCAAAGATGTGTTTCTCGTCGTTAGC	
DGAT2-N130	5F: CGACGCGTATGAAGACCCTCATAGCCGCCTACTCC	
	3R: CGGAATTCTTACTTATCGTCGTCATCCTTGTAATCACCACCACCCCAGTTTCGGACCCACTGT	
DGAT2-R123A-1	5F: CGACGCGTATGAAGACCCTCATAGCCGCCTACTCC	
	3R: GACCCACTGTGAAGCCCTGCCACCTT	
DGAT2-R123A-2	5F: AAGGTGGCAGGGCTTCACAGTGGGTC	
	3R: CGGAATTCTTACTTATCGTCGTCATCCTTGTAATCACCACCACCGTTCACCTCCAGGACCTC	
DGAT2-N120	5F: CGACGCGTATGAAGACCCTCATAGCCGCCTACTCC	
	3R: CGGAATTCTTACTTATCGTCGTCATCCTTGTAATCACCACCACCACCTTTCTTGGGTGTGTTCCAG	
DGAT2-C125	5F: CGACGCGTATGTACCCATACGATGTTCCAGATTACGCTGGTGGTGGTCAGTGGGTCCGAAACTGGGCT	
	3R: CGGAATTCTTACTTATCGTCGTCATCCTTGTAATCACCACCACCGTTCACCTCCAGGACCTC	
3×HA-DGAT2-C125	5F: CGACGCGTATGTACCCATACGATGTTCCAGATTACGCTGGTTACCCATACGATGTTCCAGATTACGCTGGTTACCCATACGATGTTCCAGATTACGCTGGTGGTGGTCAGTGGGTCCGAAACTGGGCT	
	3R: CGGAATTCTTACTTATCGTCGTCATCCTTGTAATCACCACCACCGTTCACCTCCAGGACCTC	
DGAT2-C3A	C312A	5F: TGGTTTCGCCCCAGCTATCTTCCATG
		3R: CATGGAAGATAGCTGGGGCGAAACCA
	C87‚96A	5F: GGCCGCTAGTGCCATCCTCATGTACATATTCGCTACTGATTGCTG
		3R: CAGCAATCAGTAGCGAATATGTACATGAGGATGGCACTAGCGGCC
DGAT2 121-250 aa-FLAG	5F: CGACGCGTATGGGCAGGAGGTCACAGTGG	
	3R: CGGAATTCTTACTTATCGTCGTCATCCTTGTAATCACCACCACCGCCAGGCATGGAGCTCAGA	
DGAT2 251-388 aa-FLAG	5F: CGACGCGTATGAAGAATGCAGTCACCCTGC	
	3R: CGGAATTCTTACTTATCGTCGTCATCCTTGTAATCACCACCACCGTTCACCTCCAGGACCTC	
DGAT1-FLAG	5F: CCCAAGCTTATGGGCGACCGCGGCAGCTCCCGGC	
	3R: GGAATTCTCACTTATCGTCGTCATCCTTGTAATCACCGGCCTCTGCCGCTGGGG	
ZIKV NS1	5F: CGGAATTCATGGATGTGGGGTGCTC	
	3R: CCCTCGAGCTAAGCGTAATCTGGAACATCGTATGGGTAACCACCACCTGCAGTCACCATTGACCTTAC	
ZIKV NS2B3	5F: CGGAATTCATGAGCTGGCCCCCTA	
	3R: CCCTCGAGCTAAGCGTAATCTGGAACATCGTATGGGTAACCACCACCTCCAAAAGCCGCTCCTCTT	
ZIKV NS3	5F: CGGAATTCATGAGTGGTGCTCTATGGGAT	
	3R: CCCTCGAGCTAAGCGTAATCTGGAACATCGTATGGGTAACCACCACCTCCAAAAGCCGCTCCTCTT	
ZIKV NS2B3 S135A-HA-1	5F: CGGAATTCATGAGCTGGCCCCCTA	
	3R: GGATTGGAGATCCAGCAGTTCCTGCT	
ZIKV NS2B3 S135A-HA-2	5F: AGCAGGAACTGCTGGATCTCCAATCC	
	3R: CCCTCGAGCTAAGCGTAATCTGGAACATCGTATGGGTAACCACCACCTCCAAAAGCCGCTCCTCTT	
ZIKV NS2B-NS3 Pro-HA	5F: CGGAATTCATGAGCTGGCCCCCTA	
	3R: CCCTCGAGCTAAGCGTAATCTGGAACATCGTATGGGTAACCACCACCCAGCATCGAAGGCTCG	
ZIKV NS3 Hel-HA	5F: CGGAATTCATGAAGAAGAAGCAGCTAACTGTC	
	3R: CCCTCGAGCTAAGCGTAATCTGGAACATCGTATGGGTAACCACCACCTCCAAAAGCCGCTCCTCTT	
ZIKV NS3 Pro-HA	5F: CGGAATTCATGAGTGGTGCTCTATGGGAT	
	3R: CCCTCGAGCTAAGCGTAATCTGGAACATCGTATGGGTAACCACCACCCAGCATCGAAGGCTCG	

### Generation of DGAT2- or DGAT1-knockdown cell clones

The pLKO.1-TRC plasmid (Addgene, #10878) was utilized to construct DGAT2-shRNA, DGAT1-shRNA, or control-shRNA (shCtrl, scramble sequence) to generate DGAT2- or DGAT1-knockdown cells as previously described [5]. Briefly, pLKO.1-DGAT2-shRNA, pLKO.1-DGAT1-shRNA, or pLKO.1-control-shRNA was cotransfected with psPAX2 (Addgene, #12260) and pVSVG (Addgene, #12259) into 293T cells. At 48 h after transfection, the lentivirus supernatants were collected and transduced into target cells for 24 h. After screening with puromycin for 1 week, the cells were subjected to subsequent experiments. The targeting sequences of shRNA are shown in Table 4.

**Table 4.** Oligonucleotides of DGAT2- or DGAT1-shRNA

Gene	Sequence (5′-3′)
shDGAT2-1	5F: CCGGGCTGACCACCAGGAACTATATCTCGAGATATAGTTCCTGGTGGTCAGCTTTTTG
	3R: AATTCAAAAAGCTGACCACCAGGAACTATATCTCGAGATATAGTTCCTGGTGGTCAGC
shDGAT2-2	5F: CCGGGCTACTTTCGAGACTACTTTCCTCGAGGAAAGTAGTCTCGAAAGTAGCTTTTTG
	3R: AATTCAAAAAGCTACTTTCGAGACTACTTTCCTCGAGGAAAGTAGTCTCGAAAGTAGC
shDGAT1-1	5F: CCGGCCATCCTCTTCCTCAAGCTCTCGAGAGCTTGAGGAAGAGGATGGTTTTTG
	3R: AATTCAAAAACCATCCTCTTCCTCAAGCTCTCGAGAGCTTGAGGAAGAGGATGG
shDGAT1-2	5F: CCGGGGAACATCCCTGTGCACAACTCGAGTTGTGCACAGGGATGTTCCTTTTTG
	3R: AATTCAAAAAGGAACATCCCTGTGCACAACTCGAGTTGTGCACAGGGATGTTCC
shCtrl	5F: CCGGAACGTACGCGGAATACTTCGACTCGAGTCGAAGTATTCCGCGTACGTTTTTTTG
	3R: AATTCAAAAAAACGTACGCGGAATACTTCGACTCGAGTCGAAGTATTCCGCGTACGTT

### Generation of DGAT2^RES^-, DGAT2-C3A^RES^-, DGAT2-N120^RES^-, DGAT2-R123A^RES^-, and DGAT2-C125^RES^-expressing cells

To resist *DGAT2* knockdown, the pLVEF1a-IRES-Blast-DGAT2-RES-FLAG plasmid was mutated at synonymous sites in DGAT2-shRNA targeting sequence. Then, the lentiviruses were packaged in 293T cells by transfecting pLV-DGAT2-RES-FLAG, pLV-DGAT2-C3A-FLAG, pLV-DGAT2-N120-FLAG, pLV-DGAT2-R123A-FLAG, or pLV-DGAT2-C125-FLAG with psPAX2 and pVSVG. At 48 h after transfection, the lentivirus supernatants were collected and transduced into DGAT2-knockdown cells. Then, the cells were used for subsequent experiments after screening with blasticidin for 7 d.

### Virus entry assay

Virus binding assay and internalization assay were performed as described previously [53]. Briefly, in the virus binding assay, cells were infected with ZIKV [multiplicity of infection (MOI) = 1] and incubated on ice for 45 min. In the virus internalization assay, cells were incubated with ZIKV (MOI = 1) on ice for 45 min and then incubated in ice-cold DMEM [with 2% fetal bovine serum (FBS) and 20 μM/ml NH_4_Cl] for 30 min at 37 °C. In the virus entry assay, cells were incubated with ZIKV (MOI = 1) on ice for 45 min, followed by 30-min incubation at 37 °C with 2% FBS in ice-cold DMEM. Then, the cells from virus internalization assay and virus entry assay were treated with 400 μg/ml proteinase K and kept on ice for 1 min. After 3 washes with PBS buffer, the cells were lysed in TRIzol reagent to extract total RNAs. Viral RNA levels and internal control *GAPDH* mRNA levels were detected by qRT-PCR.

### Replicon assay

The ZIKV replicon systems (pFK-SGR and pFK-SGR-GDD), obtained from G. Long’s team (Institution Pasteur of Shanghai Chinese Academy of Science), were utilized as previously described [53]. Briefly, the linearized replicon plasmids were in vitro transcribed and transfected into cells. After washing with PBS, the cells were lysed at indicated time points for luciferase activity detection. The GLOMAX 96 microplate luminometer (Promega) was used for measuring luciferase activity.

### Co-IP assay

The cells were cotransfected with indicated plasmids and harvested at 36 h after transfection for co-IP assay as described previously [51]. Briefly, the cells were lysed in radioimmunoprecipitation assay lysis buffer containing protease inhibitors and phosphatase inhibitors. Subsequently, IP was performed at 4 °C overnight with anti-HA agarose beads (HNA-25-500, NuoyiBio), and the precipitated samples were eluted in loading buffer for Western blotting.

### Immunofluorescence and confocal microscopy

The cells were fixed at 24 h after ZIKV infection (MOI = 1) and subjected to immunofluorescence as previously described [51]. In brief, the fixed cells were blocked with 5% bovine serum albumin buffer for 1 h, followed by primary antibody incubation at 4 °C overnight. After washing with PBS, the cells were incubated at room temperature with Cy3-conjugated goat anti-rabbit IgG antibody (Sigma-Aldrich) for 1 h. Subsequently, the cells were incubated with 4′,6-diamidino-2-phenylindole (DAPI) (Invitrogen) for 15 min after staining with BODIPY (Invitrogen) for 30 min. The samples were observed with a Nikon C2 microscope, and the images were analyzed with the NIS Elements software.

### Treatment of MG132 inhibitor

Indicated plasmids were transfected into cells for 24 h. Then, the cells were incubated with medium containing dimethyl sulfoxide (DMSO) or MG132 (10 μM, HY-13259, MedChemExpress) for 10 h. The cell lysates were harvested for Western blotting assay with anti-FLAG antibody.

### CHX treatment

The vector, DGAT2-WT-FLAG, or DGAT2-C125-FLAG plasmid was transfected into Huh7 cells. After 24 h of transfection, the cells were incubated with 100 μg/ml of CHX (HY12320, MedChemExpress) for 0, 1, 2, and 4 h. The cell extracts were harvested for Western blotting detection with anti-FLAG antibody.

### Detection of triglyceride content

Five 10-cm dishes of the control, DGAT1^KD^, DGAT2^KD^, and DGAT1/DGAT2 double knockdown (DGAT1^KD^DGAT2^KD^) Huh7 cells were collected for cell disruption using a sonicator (Branson). Subsequently, the amount of intracellular triglyceride was measured using a detection kit according to the manufacturer’s instructions (BC0625, Solarbio).

### OA treatment

OA (HY-N1446, MedChemExpress) was dissolved in ethanol absolute at 50 mM. Cells were infected with ZIKV (MOI = 1) for 1 h, followed by 50 μM OA addition. The cells were harvested at 24 h after infection for staining with BODIPY (green) or DAPI (blue) to visualize LD or the nucleus (scale bars, 50 μm). The supernatants were collected at 24 h after infection for plaque assay.

### Statistical analysis

The data were analyzed with GraphPad Prism 9.0 software. All the statistical analyses were performed using an unpaired, 2-tailed Student’s *t* test or analysis of variance (ANOVA) with Dunnett’s multiple comparison test. The data were presented as mean ± standard error of the mean (SEM) from at least 3 independent experiments. The differences were considered statistically significant when *P* < 0.05.

## Data Availability

All data are available in the main text or the Supplementary Materials.
