# Supplementary figures and images for: Diacylglycerol O-acyltransferase 2, a Novel Target of Flavivirus NS2B3 Protease, Promotes Zika Virus Replication by Regulating Lipid Droplet Formation
Source: Research (Wash D C). 2024 Oct 24;7:0511. doi: 10.34133/research.0511 (PMC11499588; doi:10.34133/research.0511)

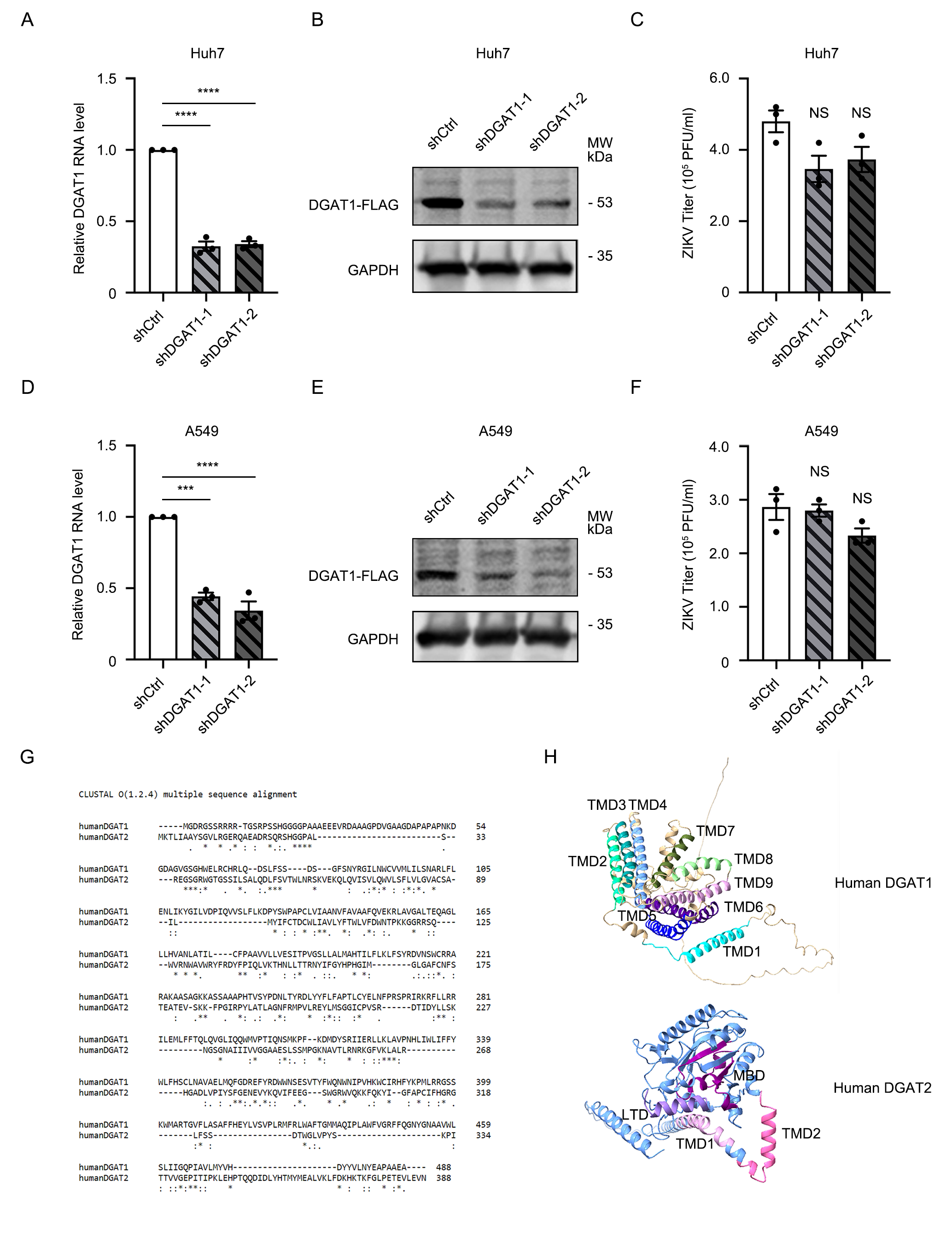

Supplement: Supplementary 1 — Figs. S1 to S6 [file research.0511.f1.zip › Supplementary Figure 1.tif]

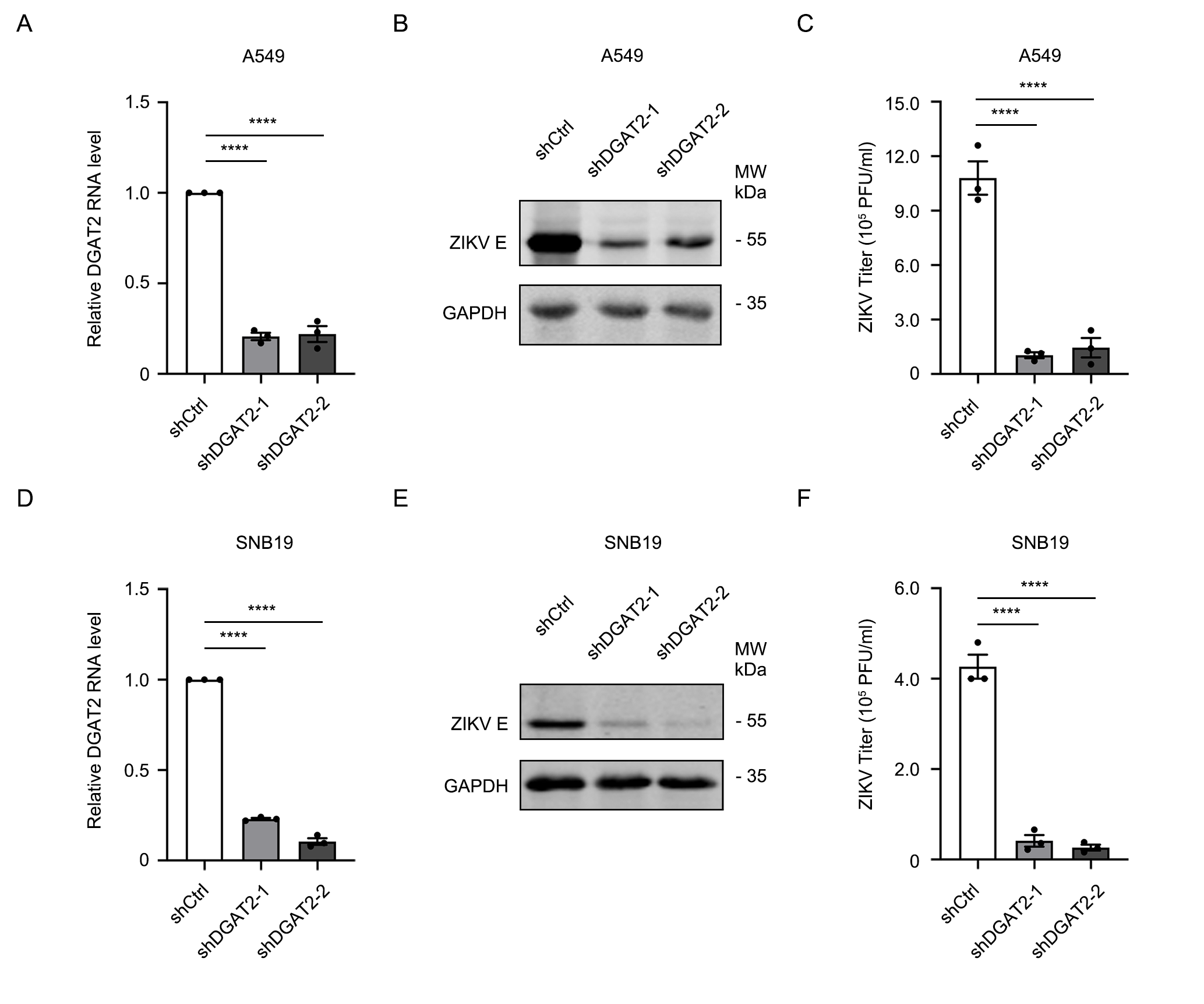

Supplement: Supplementary 1 — Figs. S1 to S6 [file research.0511.f1.zip › Supplementary Figure 2.tif]

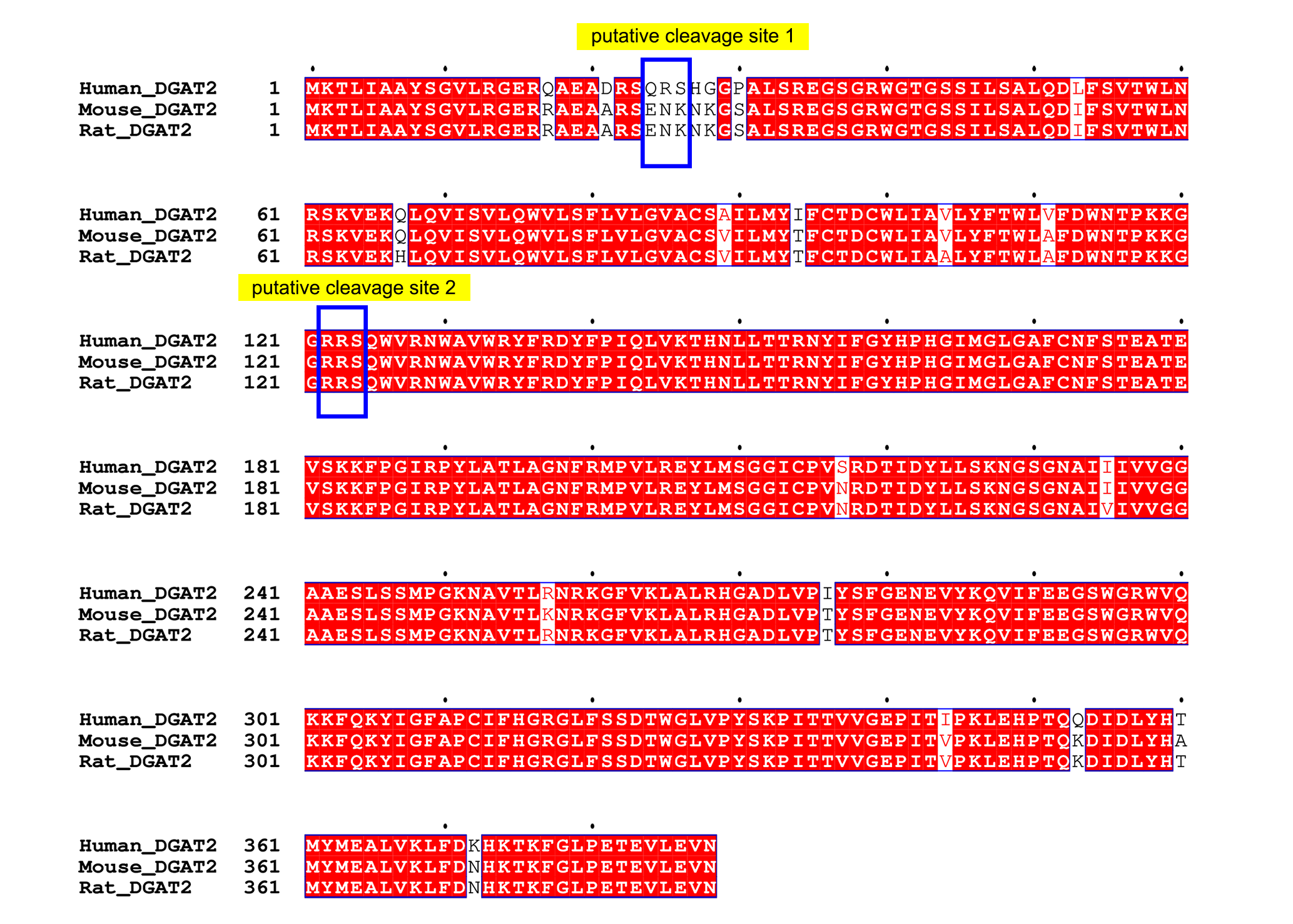

Supplement: Supplementary 1 — Figs. S1 to S6 [file research.0511.f1.zip › Supplementary Figure 3.tif]

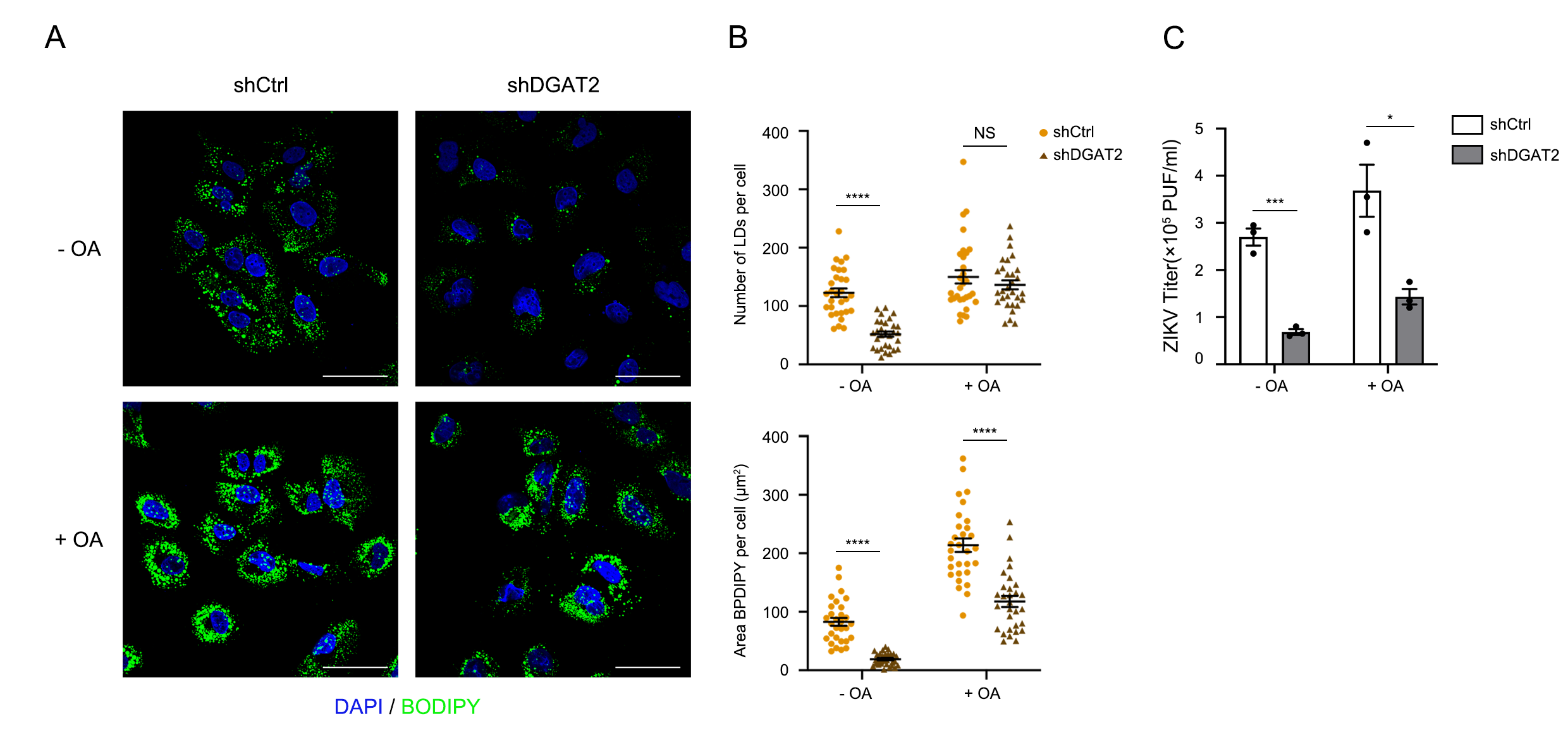

Supplement: Supplementary 1 — Figs. S1 to S6 [file research.0511.f1.zip › Supplementary Figure 4.tif]

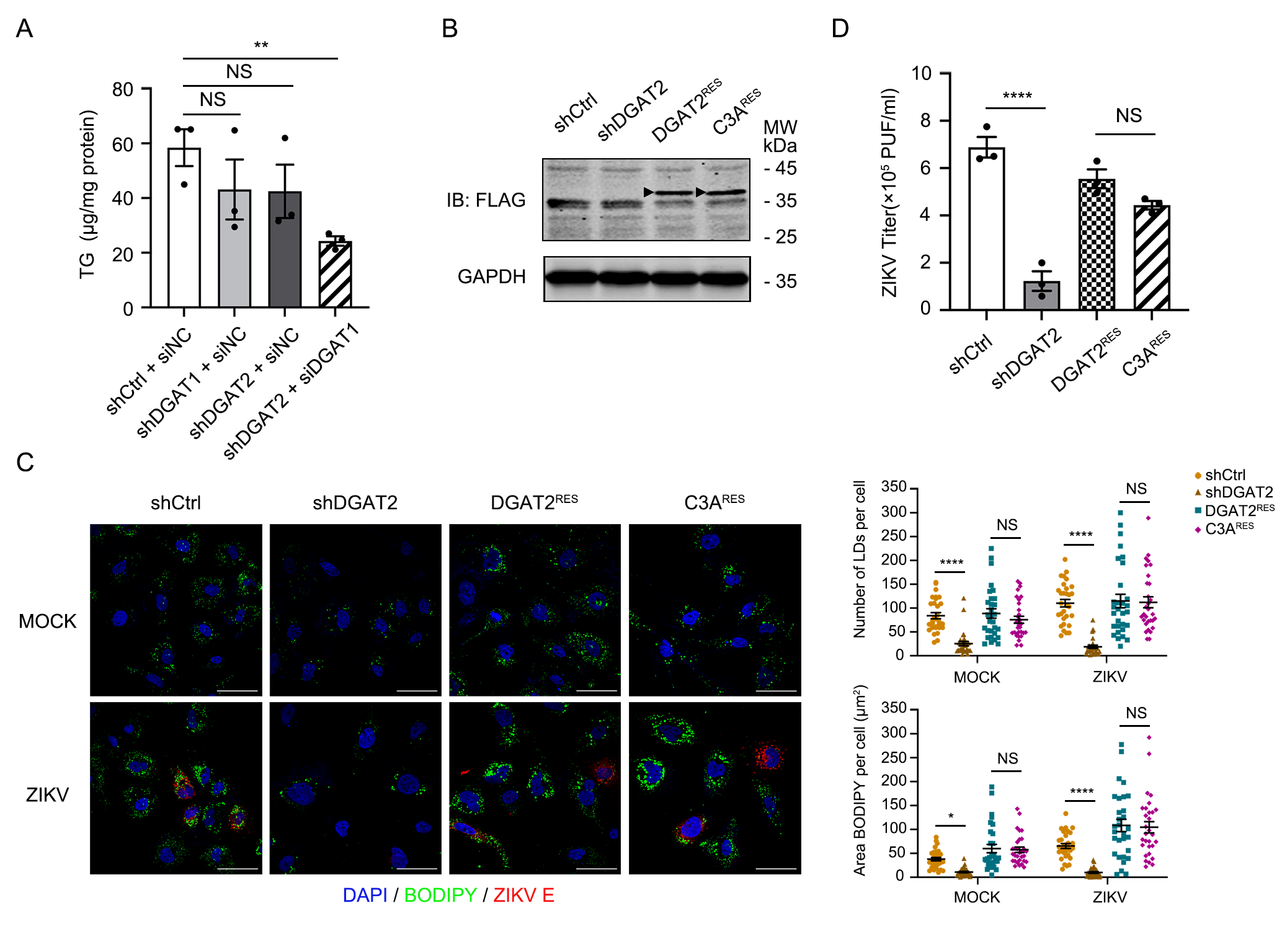

Supplement: Supplementary 1 — Figs. S1 to S6 [file research.0511.f1.zip › Supplementary Figure 5.tif]

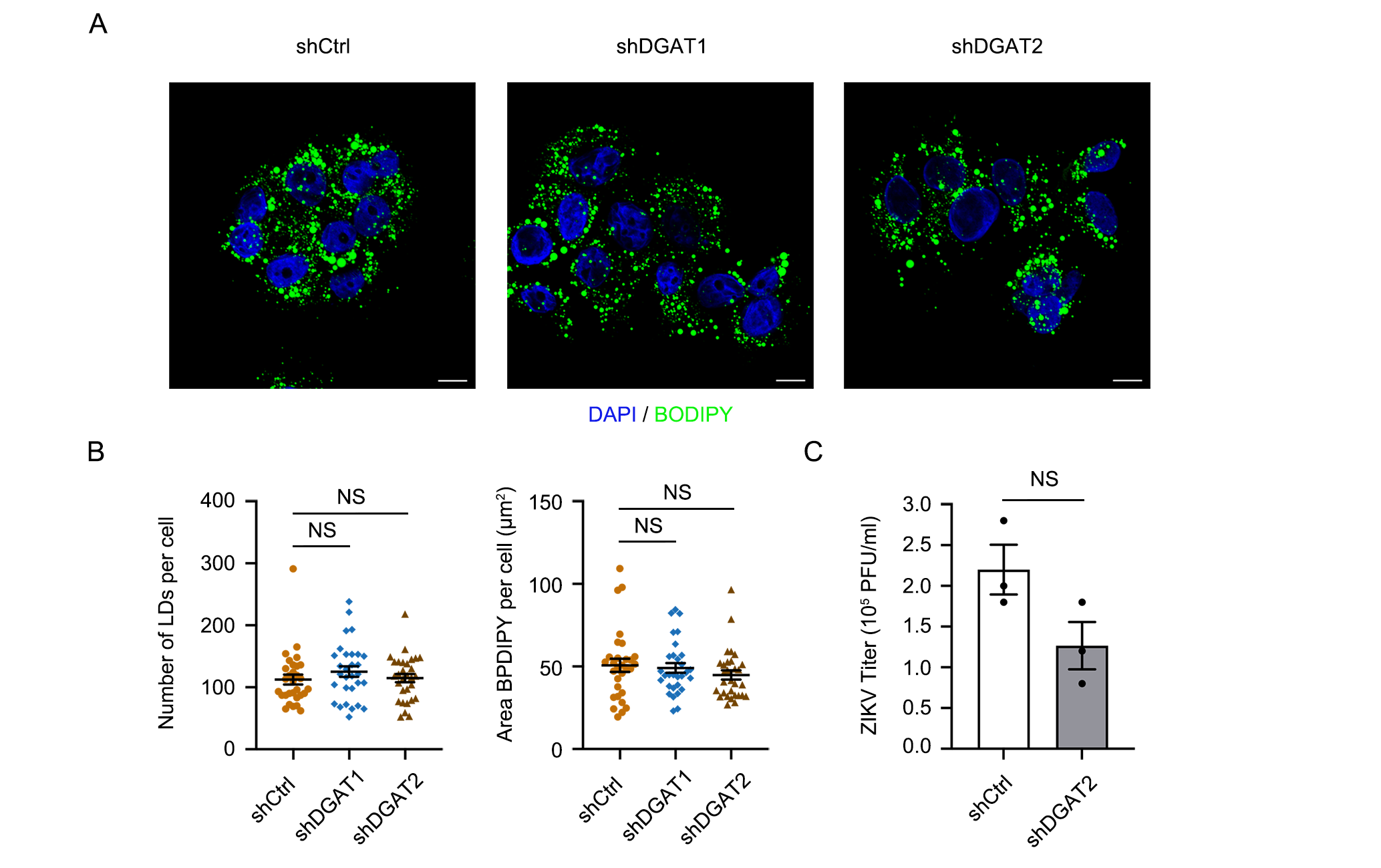

Supplement: Supplementary 1 — Figs. S1 to S6 [file research.0511.f1.zip › Supplementary Figure 6.tif]
